# Catalytic Polymerization of n‐Doped Poly(benzodifurandione) (n‐PBDF) Using Parts Per Million (ppm) Levels of Molybdenum Trioxide

**DOI:** 10.1002/anie.202510411

**Published:** 2025-07-11

**Authors:** Guangchao Liu, Uttam Pal, Sanket Samal, Michael F. Espenship, Yuanhe Li, Won‐June Lee, Lawal Adewale Ogunfowora, Liyan You, Julia Laskin, Jianguo Mei

**Affiliations:** ^1^ James Tarpo Jr. and Margaret Tarpo Department of Chemistry Purdue University West Lafayette Indiana 47907 USA

**Keywords:** Catalytic polymerization, Conducting polymers, Dimethyl sulfoxide, Molybdenum trioxide, n‐doped poly(benzodifurandione) (n‐PBDF)

## Abstract

The recent discovery of highly conductive, solution‐processable, n‐doped poly(benzodifurandione) (n‐PBDF) has significantly pushed the boundaries of organic electronics. However, to maximize its practical impact, an efficient, scalable and cost‐effective synthetic method is essential. Initially, n‐PBDF was synthesized via duroquinone‐mediated or copper‐catalyzed polymerizations, but these methods required prolonged dialysis, limiting their scalability. Our recent SeO_2_‐catalyzed polymerization improved efficiency but still necessitated centrifugation and filtration to remove solid selenium byproducts. In this work, we introduce a highly efficient molybdenum trioxide (MoO_3_)‐catalyzed polymerization of n‐PBDF. Remarkably, MoO_3_ at parts‐per‐million (ppm) concentrations achieves near‐quantitative monomer conversion (>99% by NMR), eliminating the need for purification. Kinetic studies demonstrate that this polymerization follows a chain‐growth mechanism, enabling the synthesis of high‐quality n‐PBDF polymers with controlled particle sizes and block copolymers. Mechanistic investigations reveal that MoO_3_ mediates an oxidative pathway involving dimethyl sulfoxide (DMSO), with dimethyl sulfide (DMS) identified as the reduction product. This innovation not only provides a scalable, low‐cost route to high‐quality n‐PBDF but also unlocks new synthetic opportunities, significantly expanding the synthetic toolbox for functional polymers.

## Graphical abstract

An efficient, scalable, and cost‐effective catalytic polymerization of n‐PBDF using ppm levels of MoO_3_ was developed in this study. Kinetic studies reveal that this polymerization demonstrates chain‐growth characteristics, enabling the preparation of high‐quality n‐PBDF polymers. Mechanistic investigations reveal that MoO_3_ mediates an oxidative pathway involving dimethyl sulfoxide (DMSO), with dimethyl sulfide (DMS) identified as the reduction product. This method eliminates the need for purification process, demonstrating the potential for large‐scale production of high‐quality n‐PBDF ink.

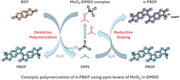

## Introduction

Conducting polymers have attracted significant attention owing to their tunable structures, solution‐processable, light‐weight and flexible, large‐area printable, tailored electrical and mechanical properties, rendering potential applications in printable electronics, energy storage systems, sensors, and bioelectronics.^[^ ^1^, ^2^, ^3^, ^4^, ^5^, ^6^, ^7^, ^8^ ^]^ Developing high‐performance and solution‐processable conducting polymers in a cost‐effective and scalable approach is crucial for conducting polymers to make a real‐world impact. High‐performance complementary p‐type and n‐type materials are required to realize various organic optoelectronic devices and integrated circuits.^[^ ^9^ ^]^ The commercially available p‐type conducting polymer ink, poly(3,4‐ethylenedioxythiophene):polystyrene sulfonate (PEDOT:PSS), has been used in numerous applications owing to its solution processability, high conductivity (>2000 S cm^−1^).^[^ ^10^, ^11^ ^]^ On the contrary, the development of n‐type conducting polymer inks lags far behind, with the poor materials stability in the ambient conditions and the cast thin films showing conductivities (typically < 100 S cm^−1^) far below PEDOT:PSS.^[^ ^9^, ^12^ ^]^ In addition, the complexity and cost‐effectiveness of synthesizing n‐type conducting polymers and their monomers limit the development of their commercialization and applications.

The recent discovery of highly conductive n‐doped poly(benzodifurandione) (n‐PBDF) with excellent stability and solution processability marks a historical milestone in the development of conducting polymers, rapidly garnering broad interest across diverse fields.^[^ ^13^, ^14^, ^15^, ^16^, ^17^, ^18^, ^19^, ^20^, ^21^ ^]^ To maximize its practical impact, the development of cost‐effective and scalable synthesis of n‐PBDF is prerequisite. Previously, n‐PBDF was prepared by either duroquinone‐mediated or copper‐catalyzed polymerizations,^[^ ^13^, ^14^ ^]^ both of which required a costly and prolonged dialysis process to remove unreacted monomers, oxidants, and other byproducts, making scalability and cost‐effectiveness potential challenges. Recently, we reported the selenium dioxide (SeO_2_) catalyzed polymerization method, in which a catalytic amount of SeO_2_ achieved high monomer conversions (>99% by NMR), eliminating the need for the dialysis process.^[^ ^22^ ^]^ This approach significantly improved scalability and cost‐effectiveness, though centrifugation and filtration were still required. Alternatively, Fabiano et al. developed a quinone‐derivative‐mediated polymerization for n‐PBDF, which retains all small molecules (including the quinone derivative and its reduced form) in the polymer thin films.^[^ ^23^ ^]^ While these residual components do not appear to adversely affect thermoelectric performance, the method suffers from limited generalizability for other application scenarios. In other words, these methods still do not meet the criteria of scalability and cost‐effectiveness.

**Figure 1:**
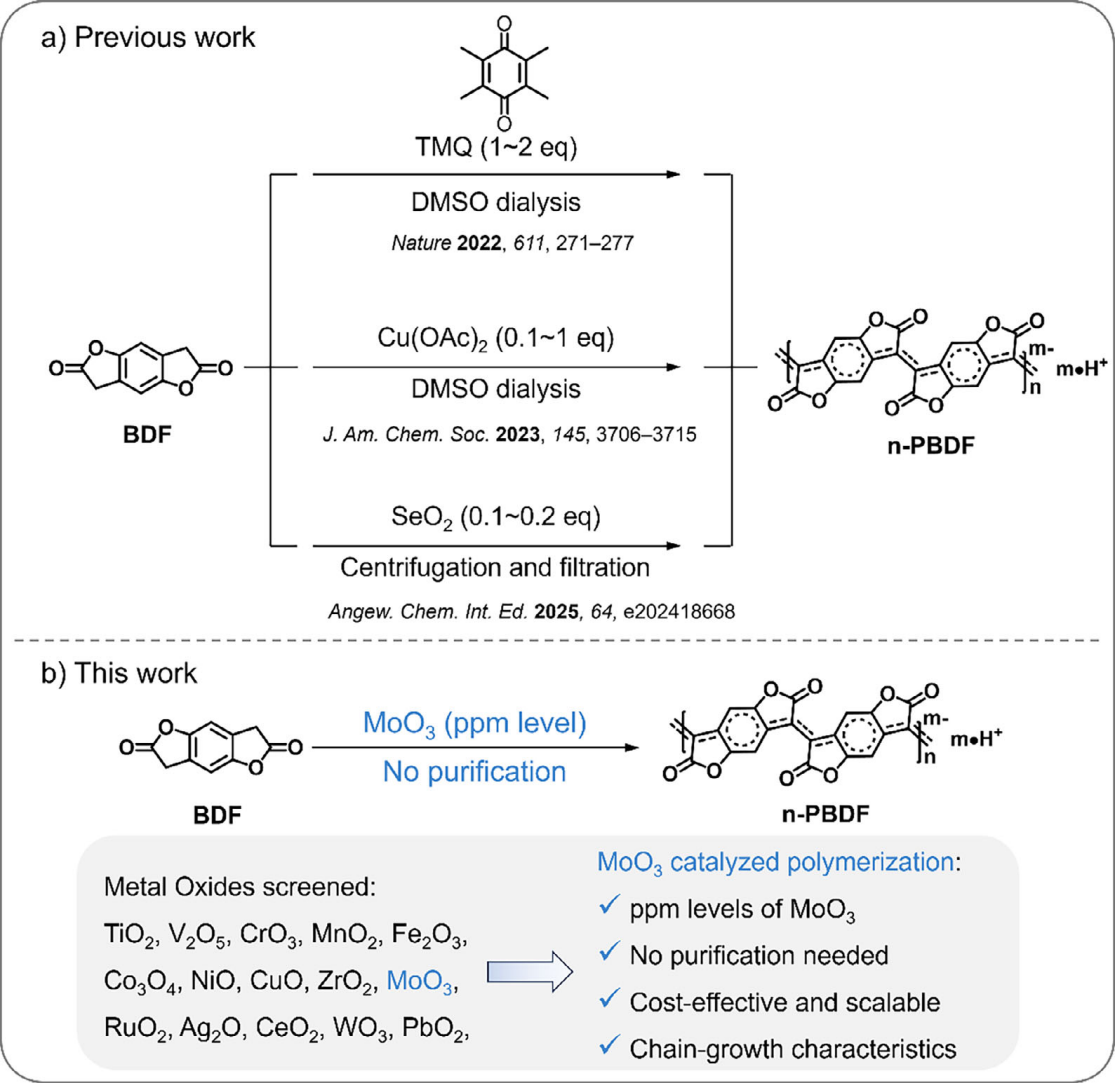
Schematic diagram of n‐PBDF synthesis using a) TMQ, Cu(OAc)_2_, and SeO_2_ in previous work, and b) MoO_3_ in this work.

Dimethyl sulfoxide (DMSO) is widely recognized as an effective oxidant in reactions such as the Swern oxidation, though these transformations typically require stoichiometric activating agents (e.g., acylating agents include acyl chlorides and anhydrides, SO_3_‐pyridine, carbodiimide, P_2_O_5_).^[^ ^24^, ^25^, ^26^, ^27^, ^28^, ^29^, ^30^, ^31^, ^32^, ^33^, ^34^ ^]^ To address this limitation, catalytic systems for alcohol oxidation—mediated by metal oxides—have been developed, eliminating the need for stoichiometric activators.^[^ ^35^, ^36^ ^]^ In the synthesis of n‐PBDF, DMSO serves dual roles: as the solvent and as a mediator of benzodifurandione (BDF) monomer tautomerization. However, its capacity to act as an oxidant in the oxidative polymerization process has not been demonstrated. In this study, we explored whether metal oxides‐DMSO complexes can catalyze the oxidative polymerization of n‐PBDF. We discovered the catalytic polymerization of n‐PBDF by using ppm level MoO_3_ (Figure 1). This new polymerization method offers four key advantages: (1) exceptional monomer conversion (>99% by NMR) achieved using ppm‐level MoO_3_; (2) production of high‐quality, controlled particle sizes n‐PBDF polymer ink with high conductivity (>2000 S cm^−1^); (3) elimination of purification steps due to near‐quantitative monomer conversion and minimal residual catalyst (ppm level); and (4) cost‐effectiveness and scalability, enabled by the use of inexpensive MoO_3_ catalyst at ppm concentrations and the absence of post‐synthesis purification. Furthermore, the MoO_3_ catalyzed polymerization is featured as a controlled chain growth polymerization, enabling the synthesis of high‐quality n‐PBDF polymers with controlled particle sizes and block copolymers. Finally, we presented the MoO_3_ mediated dimethyl sulfoxide (DMSO) oxidative pathway, in which dimethyl sulfide (DMS) was detected as the reduction product.

## Results and Discussion

### MoO_3_ Catalyzed Polymerization of n‐PBDF

We screened a series of metal oxides (TiO_2_, RuO_2_, WO_3_, MoO_3_, CrO_3_, Fe_2_O_3_, CuO, Ag_2_O, V_2_O_5_, MnO_2_, Co_3_O_4_, NiO, ZrO_2_, CeO_2_ and PbO_2_) for the catalytic polymerization of n‐PBDF (Figure 1b). BDF monomers were polymerized with catalytic amount (0.1 equiv.) of metal oxides at 100 °C for 24 h (Figure S1 and Table S1). To monitor the polymerization, the ultraviolet–visible–near infrared (UV–vis–NIR) absorption spectra of obtained mixtures were measured (Figure S1). The mixtures obtained from the TiO_2_, RuO_2_, and WO_3_ mediated reactions show characteristic absorption curve of n‐PBDF, in which strong polaron and bipolaron absorption bands are observed in the NIR region, and their thin films exhibit high electrical conductivities. While in CrO_3_, Fe_2_O_3_, CuO, and Ag_2_O mediated reactions, the obtained mixtures exhibit weak polaron and bipolaron absorption bands in the NIR region, and strong neutral peaks (400∼600 and 600∼1000 nm),^[^ ^18^ ^]^ suggesting that the obtained products are low molecular weight oligomers with low doping levels. However, in the UV–vis–NIR absorption spectra obtained from reactions mediated by V_2_O_5_, MnO_2_, Co_3_O_4_, NiO, ZrO_2_, CeO_2_ and PbO_2_, no polaron and bipolar absorption bands are observed in the NIR region, but absorption peaks are present in the visible region, indicating that the resulting products are undoped, low molecular weight species.

Notably, among all metal oxide‐catalyzed polymerizations tested, only MoO_3_ produced an insoluble gel—a hallmark of efficient chain propagation. By optimizing reaction conditions (shortened time and reduced MoO_3_ loading to 0.005 equiv.), we achieved soluble n‐PBDF products (Table S2). The resulting inks exhibit characteristic n‐PBDF absorption profiles (Figure S1d), and their thin films demonstrate high electrical conductivities exceeding 2000 S cm^−1^ (Table S2). The polymerization was successfully catalyzed by MoO_3_ at ultralow loadings (0.005 equivalents), prompting us to explore further catalyst reduction. Complete monomer conversion with negligible catalyst residues could theoretically eliminate purification—a key practical advantage we wish to accomplish. However, in TiO_2_‐, WO₃‐, and RuO_2_‐catalyzed systems, polymerization efficiency sharply declined at reduced loadings. This stark contrast in catalytic performance led us to focus exclusively on MoO_3_ for subsequent studies.

Given that polymerization proceeds efficiently with MoO_3_ loadings as low as 0.005 equivalents (eq), we sought to push this catalytic system to even lower catalyst concentrations. However, accurately weighing and transferring sub‐milligram quantities of MoO_3_—particularly for small‐scale reactions—posed significant practical challenges. To overcome this, we developed a reproducible protocol: MoO_3_ was first fully dissolved in DMSO to prepare standardized stock solutions, ensuring precise catalyst delivery at ultralow loadings.^[^ ^37^ ^] 1^H NMR spectra were recorded during the polymerization to monitor the monomer conversion and the degree of polymerization (Figures S2–S11). We first optimized monomer concentration for polymerizations using 0.001 eq of MoO_3_ at 100 °C (Table 1, entries 1–4; Figures S2–S6). At high monomer concentrations (>7.5 mg mL^−1^), rapid gel formation hindered chain propagation, leading to incomplete monomer conversion. Reducing the monomer concentration below 7.5 mg mL^−1^ prevented gelation, maintaining a homogeneous solution throughout the reaction and enabling full conversion. Next, we evaluated polymerization temperature (Table 1, entries 4–8; Figures S7–S11). Increasing the temperature accelerated reaction kinetics but caused a moderate reduction in polymer conductivity. Conversely, lower temperatures (at 80 °C) dramatically slowed polymerization, requiring extended reaction times to achieve full monomer conversion. Building on optimized conditions (5 mg mL^−1^ monomer concentration, 100 °C), we further reduced MoO_3_ loading to 100 ppm (Table 1, entry 9). Despite this ultralow catalyst concentration, full monomer conversion was maintained, though polymer conductivity showed a moderate reduction to ∼1600 S cm^−1^. To assess scalability, we conducted 1‐liter‐scale polymerizations at 100 and 10 ppm MoO_3_ loadings. Remarkably, both scales achieved complete monomer conversion, demonstrating robust catalytic efficiency even at trace catalyst levels. The details can be found in the “scale up” session.

**Table 1:** Investigation of polymerization conditions

Entry	MoO_3_ ^a)^ (equiv.)	Monomer Concentration (mg mL^−1^)	Temperature (°C)	Time (h)^b)^	Monomer Conversion (%)^c)^	Appearance	Conductivity (S cm^−1^)	Conductivity After Dialysis (S cm^−1^)
1	0.001	15	100	7	33	gel	—^d)^	1816 ± 25
2	0.001	10	100	9	53	gel	—^d)^	1896 ± 63
3	0.001	7.5	100	15	>99	viscous solution	1976 ± 75	2179 ± 80
4	0.001	5	100	15	>99	solution	2075 ± 130	2112 ± 52
5	0.001	5	80	72	>99	solution	1983 ± 22	2247 ± 14
6	0.001	5	120	4	>99	solution	1434 ± 107	1549 ± 29
7	0.001	5	140	1.67	>99	solution	1067 ± 7	1260 ± 29
8	0.001	5	160	0.67	>99	solution	817 ± 22	833 ± 33
9	0.0001	5	100	36	>99	solution	1696 ± 82	1815 ± 56
10^e)^	0.0001	5	100	60	>99	solution	1399 ± 31	1588 ± 65
11^e)^	0.00001	5	100	240	>99	solution	567 ± 16	569 ± 8

^a)^ MoO_3_ was fully dissolved in DMSO to prepare its DMSO solution for polymerization.

^b)^ Polymerization time depends on when the gels were formed or on complete monomer conversion if no gels formed.

^c)^ Obtained from ^1^H NMR.

^d)^ Not measured because unreacted monomers remained in unpurified inks.

^e)^ Scale‐up polymerization (1 L) without further purification. All other polymerizations were in 10 mL volumes. All conductivities were obtained by the four‐point probe method.

### Polymerization Kinetics Study

**Figure 2:**
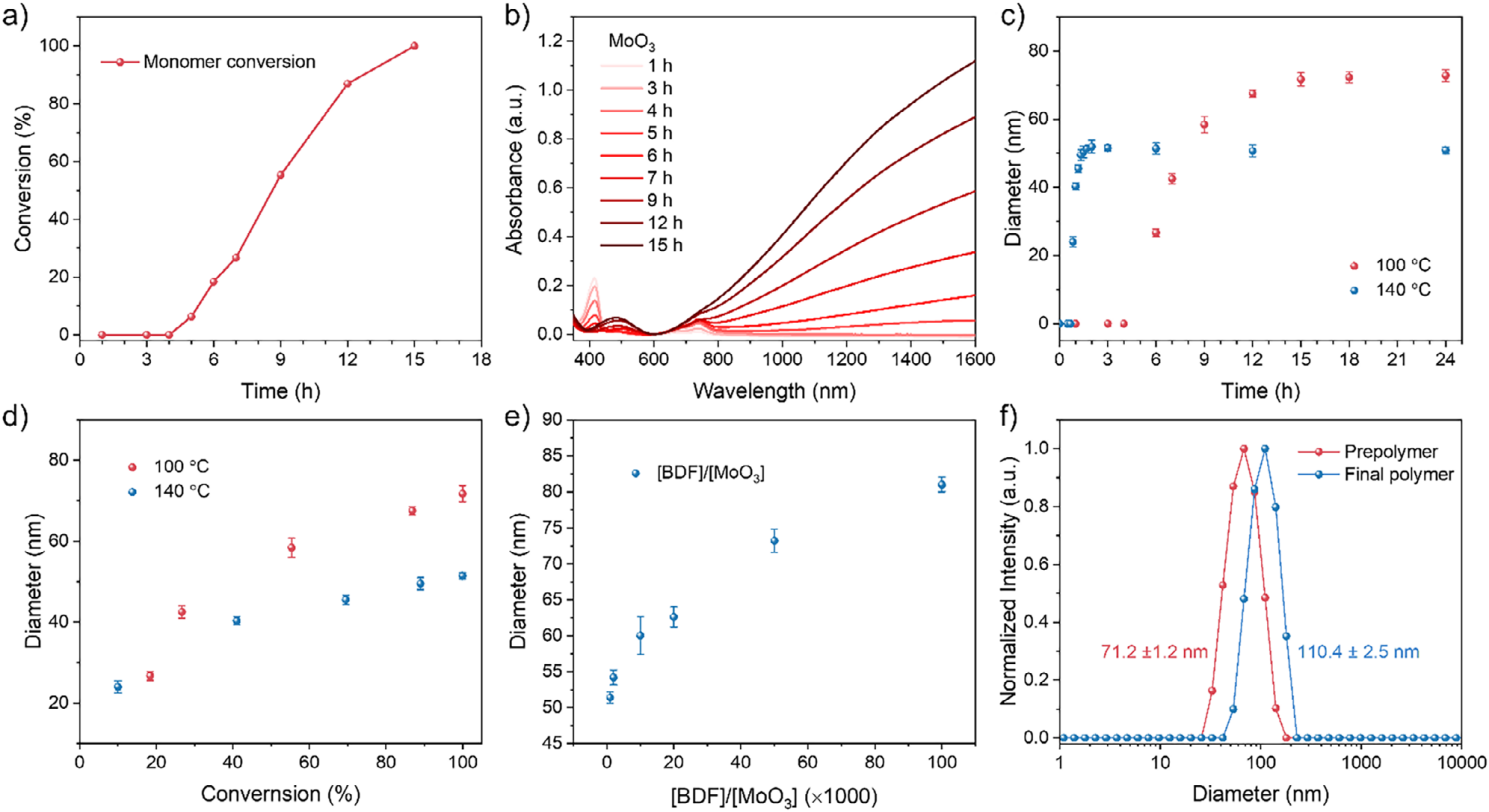
Kinetics study of MoO_3_ catalyzed polymerization of n‐PBDF. a) Time‐dependent conversion of BDF monomer, b) Time‐dependent UV–vis–NIR spectra, c) Time‐dependent hydrodynamic diameter evolution, d) Hydrodynamic diameters as a function of monomer conversions (Polymerization conditions: 5 mg mL^−1^ BDF monomer with 0.001 equiv. MoO_3_). e) Hydrodynamic diameters as a function of [BDF]/[MoO_3_] ratios. f) Hydrodynamic diameter evolution in monomer addition experiment.

To elucidate the polymerization mechanism and kinetics, we conducted time‐dependent studies using ¹H NMR spectroscopy, UV–vis–NIR spectroscopy, and dynamic light scattering (DLS). ¹H NMR revealed near‐linear monomer consumption after an initial activation, achieving full conversion within 15 h (Figures 2a, S5, and S6d). Concurrently, UV–vis–NIR spectra displayed progressive intensification of polaron and bipolaron absorption bands (Figure 2b), consistent with the gradual formation of n‐PBDF. DLS further corroborated these kinetics: hydrodynamic diameters increased steadily during polymerization (Figures 2c and S12; Table S3), suggesting progressive chain propagation and molecular weight growth. Critically, particle size plateaued immediately after monomer depletion, even with prolonged reaction times (Figures 2c and S12; Table S3). This termination behavior starkly contrasts with step‐growth or polycondensation mechanisms, where chain coupling persists after the monomer consumption. At elevated temperatures (140 °C), polymerization accelerated but yielded smaller hydrodynamic diameters compared to 100 °C (Figures 2c, S12–S13; Tables S3–S4). To elucidate whether chain‐growth polymerization takes place in this polycondensation, we investigated the relationship between the conversion of the monomer, the feed ratio of monomer to MoO_3_ catalyst, and the length of the formed n‐PBDF polymer, which can be qualitatively indicated with hydrodynamic diameter. As shown in Figure 2d, at both 100 °C and 140 °C, the hydrodynamic diameter of n‐PBDF polymers increased significantly with respect to the increase of monomer conversion. The length of the polymer also increase significantly with the increase of the [BDF]:[MoO_3_] feed ratio (Figure 2e, Figure S14 and Table S5). These results suggest that MoO_3_ catalyzed polymerization exhibits characteristics of chain‐growth polymerization and enables the synthesis of n‐PBDF polymers with controlled particle sizes.

The chain‐growth feature of the polymerization was also examined by a “monomer‐addition” experiment (Figure 2f, and see Section 4 in Supporting Information for more details), in which a fresh batch of BDF monomer was added into the prepolymer solution, and the newly added monomer was fully converted after the mixture continued stirring for another 6 h. As shown in Figures 2f and S15, the hydrodynamic diameter of the product clearly increased to 110.4 from 71.2 nm of the prepolymer. To verify this result, three control experiments were carried out simultaneously based on the same prepolymer. In the first control experiment, a fresh batch of monomer and MoO_3_ were added into the prepolymer solution, and the newly added monomer was also fully converted after stirring for 6 h. The hydrodynamic diameter of the product was almost the same as that obtained from the “monomer‐addition” experiment without the addition of fresh MoO_3_. This result suggests that even with the addition of fresh catalyst, the newly added monomers preferentially polymerize on the prepolymer chains rather than restarting the formation of new polymer chains. In the second control experiment, the prepolymer solution continued stirring for another 6 h and hydrodynamic diameter showed almost no change, which was consistent with the results in Figure 2c. In the third control experiment with only pure DMSO added, the result was similar to the second control experiment. All these results indicate that MoO_3_ catalyzed polymerization has chain‐growth polymerization characteristics and demonstrates potential for the synthesis of block copolymers by sequential addition of monomers.

### Mechanistic Understanding of the Catalytic Polymerization

**Figure 3:**
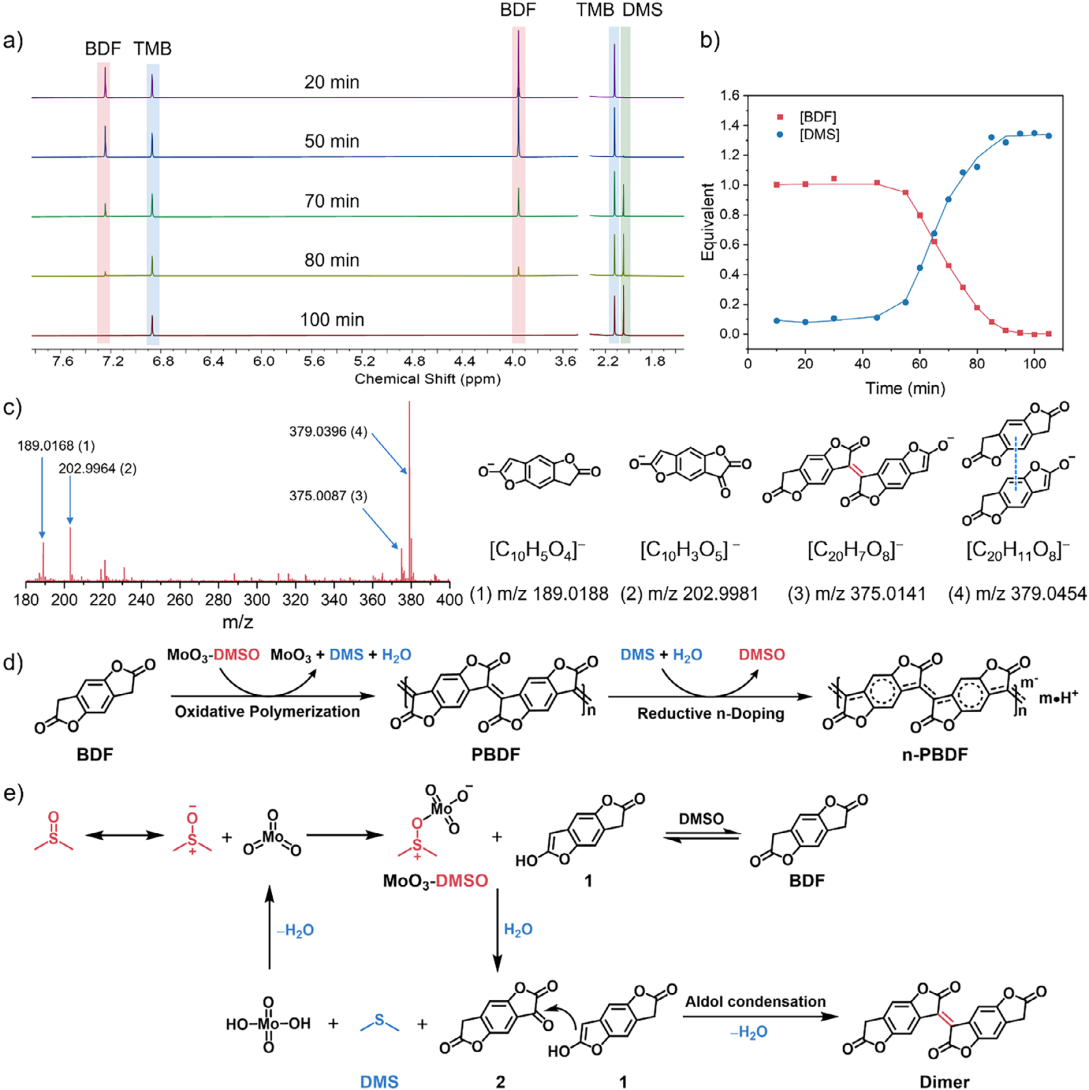
Mechanistic understanding of MoO_3_ catalyzed polymerization of n‐PBDF. a) ^1^H NMR monitoring of the polymerization process for the gradually disappearance of BDF and appearance of DMS during the polymerization process. 1,2,4,5‐Tetramethylbenzene (TMB) was used as a reference. b) The evolution of BDF and DMS during the polymerization process. c) ESI‐MS of the mixtures from 0.001 eq MoO_3_ catalyzed polymerization in 1 h and the detected intermediates and products. d) The proposed mechanisms of oxidative polymerization of BDF and reductive n‐doping of PBDF. e) The MoO_3_–DMSO complex mediated dimerization process.

Kinetic studies indicate that MoO_3_ serves as an “initiator” in the polymerization reaction. To determine whether MoO_3_ also functions as an oxidant in the oxidative polymerization mechanism, we analyzed the reaction orders of BDF and MoO_3_, which provide critical insights into the reaction pathway. During detailed kinetic studies in DMSO, time‐dependent ¹H NMR spectra revealed two key observations (Figures 3a and S16): (1) gradual depletion of BDF monomer peaks and (2) emergence of a new peak at 2.05 ppm. This peak, absent in pre‐reaction spectra, excludes the possibility of solvent‐derived interference, confirming its origin as a reaction byproduct. The temporal correlation (Figure 3b) between peak growth and polymerization progress suggests that the compound arises from DMSO during the reaction. Comparison with literature chemical shifts (*δ* = 2.05–2.10 ppm) confirms its assignment to dimethyl sulfide (DMS), establishing DMSO as the oxidant in the MoO_3_‐mediated mechanism.^[^ ^38^ ^]^ It was reported that MoO_3_ dissolves in DMSO to give a complex and this MoO_3_‐DMSO complex can catalyze oxidation of alcohol to carbonyl compound.^[^ ^35^, ^37^ ^]^ Thus, we confirm that PBDF forms via MoO_3_‐catalyzed oxidative polymerization in DMSO, during which DMSO is reduced to DMS. The resulting DMS could subsequently mediate the in situ reductive doping of PBDF, yielding n‐PBDF (Figure 3d). To further prove this possible doping pathway, the dedoped PBDF thin films were immersed in the DMS solutions and exhibited a gradual recovery of polaron and bipolaron absorption bands in the NIR region, along with a decrease in the neutral peaks at 895 and 500 nm, providing evidence of DMS re‐doping PBDF back to n‐PBDF (Figure S17).

To comprehend the detailed oxidative polymerization pathway of MoO_3_ catalyzed polymerization, high‐resolution electrospray ionization mass spectrometry (ESI‐MS) of the mixtures at the initial stage of the polymerizations were performed (Figures 3c and S18–S19). The ESI‐MS results show that in the early stage of polymerization, in addition to the BDF monomer (*m/z* 189.0168), the diketone intermediate (*m/z* 202.9964) and the dimer (*m/z* 375.0087) were also detected. This result suggests that the oxidative polymerization process most likely undergoes the diketone intermediate and aldol condensation pathway.^[^ ^22^ ^]^ To further investigate the polymerization mechanism, a commonly used radical trapping agent, *N*‐*tert*‐Butyl‐α‐phenylnitrone (PBN), was added into the reaction (see Figures S20–S22 for more details). However, it was found that no spin adducts with PBN participating were observed in ESI‐MS, and no electron paramagnetic resonance (EPR) signal was observed in reactions with or without addition of PBN. As a result, the polymerization proceeded relatively smoothly, the monomer was fully consumed, and the UV–vis–NIR absorption spectrum of the obtained solution shows a similar absorption curve to that of n‐PBDF, which exhibits strong polaron and bipolaron absorption bands in the NIR region (Figure S22). All these results further prove that the oxidative polymerization process undergoes the diketone intermediate and aldol condensation pathway, rather than the radical procedure observed in TMQ and Cu(OAc)_2_ systems.^[^ ^13^, ^14^ ^]^ Therefore, as shown in Figure 3e, we propose that the dimerization process involves the MoO_3_–DMSO complex mediated oxidation of the BDF monomer to form a diketone intermediate, which is then followed by the aldol condensation with another BDF monomer to form the dimer. The detailed proposed mechanisms for the MoO_3_–DMSO complex mediated dimerization process were presented in Figure S23. With successive MoO_3_–DMSO complex mediated oxidation and aldol polycondensation processes, the growing polymer chain propagating which undergoes in situ reductive doping by DMS to yield n‐PBDF. With the mechanistic understanding of activated‐DMSO oxidation pathway, we further demonstrated the potential of other DMSO activators (e.g., carbodiimide, SO_3_‐pyridine, P_2_O_5_, acyl chlorides) in mediating the oxidative polymerization of BDF and proposed a general DMSO oxidation pathway of BDF monomers by using DMSO‐activating agents (Figures S24–S25).

**Figure 4:**
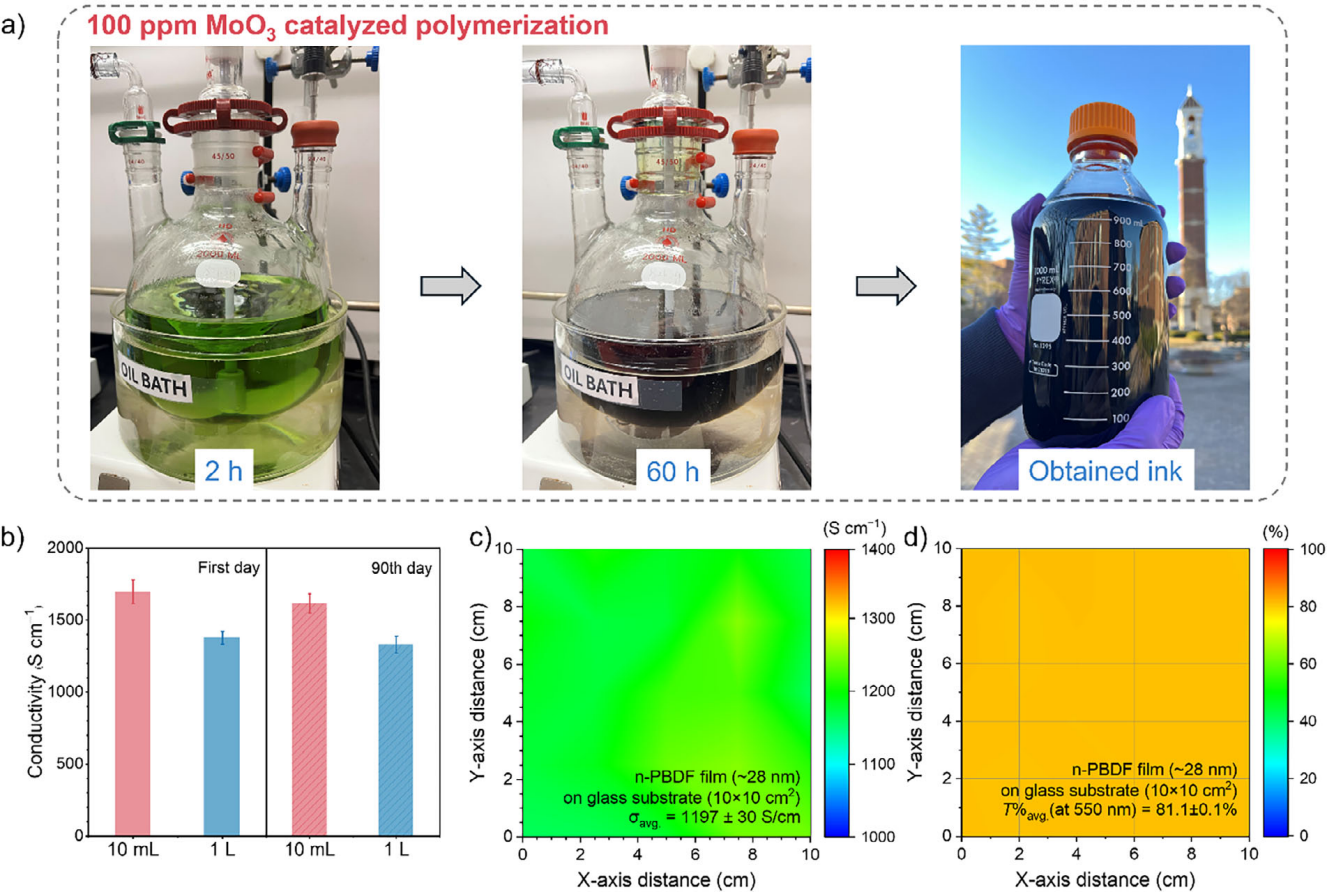
Scale‐up synthesis of n‐PBDF by MoO_3_ catalyzed polymerization. a) Scale‐up synthesis (1 L) of n‐PBDF by using 100 ppm MoO_3_. b) Scalability and stability of n‐PBDF ink by using 100 ppm MoO_3_ catalyzed polymerization. c) The conductivity mapping of the large‐area n‐PBDF thin film. d) The transmittance mapping of the large‐area n‐PBDF thin film.

We observed an “initiation period” in the MoO_3_‐catalyzed polymerization, during which no significant monomer conversion was detectable by ¹H NMR in the early reaction stage (Figures 2a and S2–S11). This initiation phase varied with polymerization temperature and catalyst loading. Despite the absence of detectable monomer consumption, spectroscopic and ESI‐MS data revealed active early‐stage processes: the changes in the UV–vis–NIR absorption peaks and the solution′s color (from colorless to green) indicated the initial progression of the reaction (Figures 2b, 4a, and S28), while ESI‐MS confirmed the formation of diketone and dimer intermediates. The low MoO_3_ loading (ppm‐level) limited dimer formation initially, resulting in concentrations too low for ¹H NMR detection. These early dimers formed charge‐transfer complexes (CTCs) with excess BDF monomers (observed as *m/z* 565.0400 in ESI‐MS, Figure S20).^[^ ^39^ ^]^ We hypothesize that CTC formation reduces the dimer's oxidative activity, hindering doping and delaying subsequent polymerization (Figure S26). However, as polymer chains elongate, they become more susceptible to MoO_3_‐DMSO oxidation and DMS‐mediated doping. This creates a self‐accelerating mechanism, ultimately driving the reaction to completion.

### Scale‐Up Synthesis of n‐PBDF Using ppm‐Level MoO_3_

To maximize its practical impact, a scalable and cost‐effective synthetic method is essential for mass production of n‐PBDF. Using ppm‐level MoO_3_ loading enables a complete monomer conversion (Table 1), eliminating the need for purification. Building on the success of 100 ppm MoO_3_ in catalyzing polymerization (Table 1, entry 9), we explored large‐scale synthesis with 100 ppm and lower loadings. Figure 4a illustrates the scale‐up synthesis process of n‐PBDF using 100 ppm MoO_3_. Stirring at 100 °C for 60 h resulted in complete monomer conversion, confirmed by TLC and ¹H NMR (Figure S27). This process yielded high‐quality n‐PBDF ink at a 1 L scale with a conductivity of up to 1400 S cm^−1^, requiring no purification (Table 1, entry 10). The 100‐fold scale‐up produced ink with conductivity comparable to the 10 mL batch (Table 1, entries 9–10; Figure 4b). Both inks exhibited excellent ambient stability, retaining over 95% of their initial conductivity after 90 days of air exposure (Figure 4b). To assess ink quality for large‐scale applications, we fabricated a large‐area transparent organic conductor (TOC) film via spray‐coating onto glass substrate. The TOC film demonstrated a high average conductivity of 1200 S cm^−1^ (four‐point probe) and over 80% transparency at 550 nm (peak human eye sensitivity), with an average sheet resistance of 298 Ω sq^−1^ (Figures 4c,d). We further scaled the synthesis to 1 L using 10 ppm MoO_3_ (Figure S28). While polymerization was slower than with 100 ppm, full monomer conversion occurred within 10 days (Figure S29). Thus, we demonstrate that high‐quality n‐PBDF ink is scalable through this efficient, ppm‐level MoO_3_‐catalyzed method. This cost‐effective, scalable approach—requiring no purification—will accelerate n‐PBDF adoption in large‐scale printed electronics and beyond.

### Generalization and Application of MoO_3_ Catalyzed Polymerization

To explore the versatility of the MoO_3_ catalyzed polymerization in the synthesizing of relevant n‐type conjugated polymers, the sulfur derivatives of BDF monomer, namely 3,7‐dihydrobenzo[1,2‐b:4,5‐b']dithiophene‐2,6‐dione (BDT) was polymerized with 0.001 eq MoO_3_. The BDT monomer was completely polymerized after 12 h, as monitored by ^1^H NMR (Figure S30). This prepolymer can continue to propagate by adding the second batch of BDT monomer, and the newly added BDT monomer was rapidly and completely polymerized after 1 h. This phenomenon aligns with the “monomer‐addition” experiment in the polymerization of BDF monomer: the newly added monomers preferentially continue polymerizing on the prepolymer chains due to higher reactivity of the prepolymer. This result demonstrated that MoO_3_ catalyzed polymerization is applicable to n‐PBDF derivatives, further proving the generality and chain‐growth polymerization characteristics of this method.

**Figure 5:**
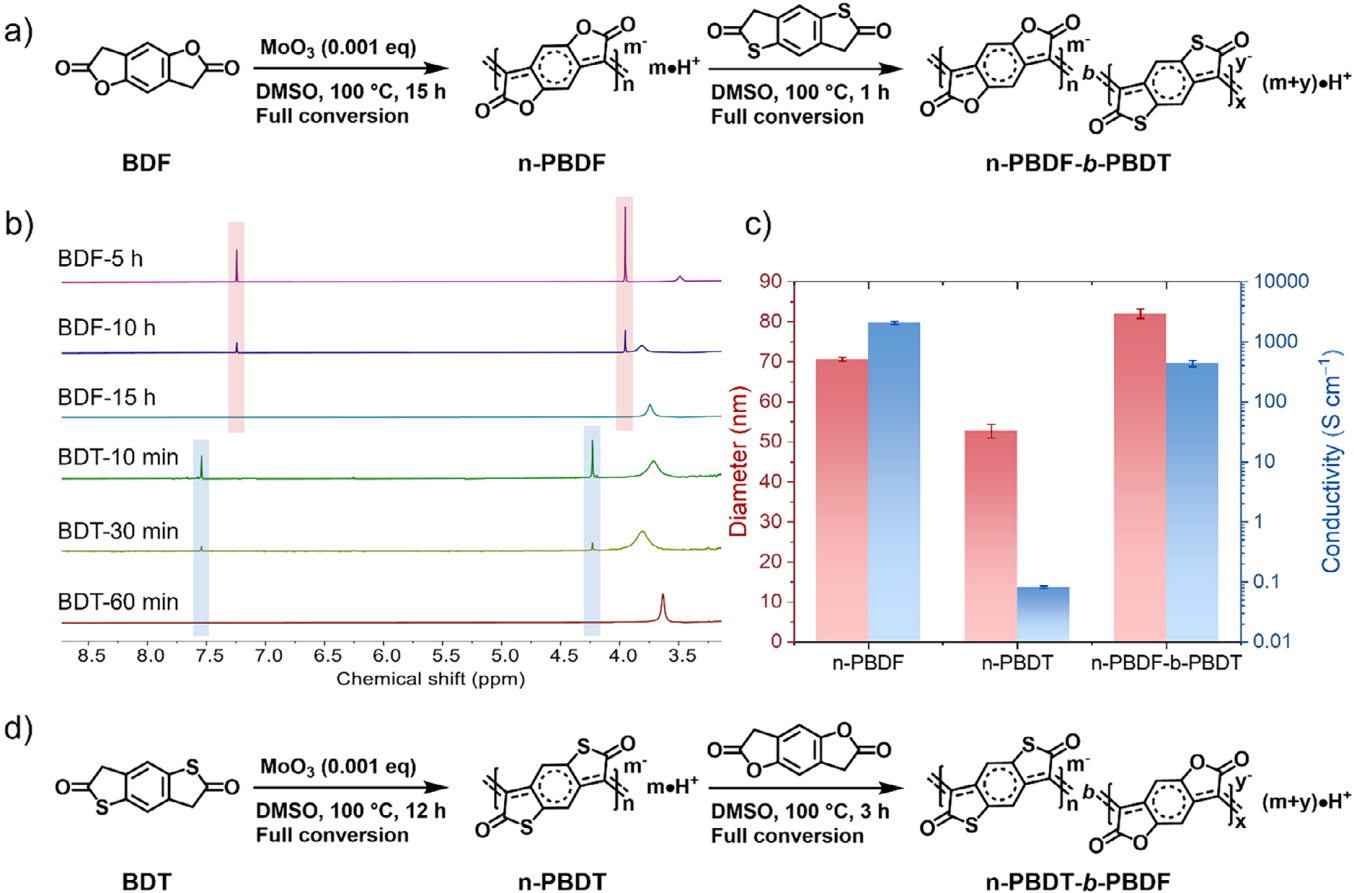
Generalization and application of MoO_3_ catalyzed polymerization. a) Synthesis of diblock copolymer n‐PBDF‐*b*‐PBDT by sequential addition of monomers. b) ^1^H NMR monitoring of the polymerization process of block copolymer n‐PBDF‐*b*‐PBDT. The proton peaks of BDF and BDT are marked as red and blue, respectively. c) Hydrodynamic diameters and conductivities characterization of block copolymer n‐PBDF‐*b*‐PBDT and its corresponding homopolymers. d) Synthesis of diblock copolymer n‐doped PBDT‐*b*‐PBDF by sequential addition of monomers.

With the kinetic and mechanistic understanding of MoO_3_ catalyzed polymerization, we proceeded to explore its potential in synthesizing block copolymers. As shown in Figure 5, diblock copolymers were synthesized by using sequential addition of BDF and BDT monomers. n‐PBDF prepolymer was prepared when BDF monomer was fully polymerized in the presense of 0.001 eq MoO_3_ at 100 °C for 15 h, then BDT monomer was added into the prepolymer solution and fully converted after 1 h, monitored by ^1^H NMR (Figure 5b). The newly added BDT monomer was rapidly and completely polymerized within 1 h, which was much faster than its homopolymerization (12 h). The hydrodynamic diameters and conductivities were characterized, with the results summarized in Figure 5c and Table S6. The hydrodynamic diameter of block copolymer n‐PBDF‐*b*‐PBDT increased to 80.3 from 69.4 nm of the prepolymer n‐PBDF, indicating that the BDT monomer was polymerized on the n‐PBDF prepolymer chain. The conductivity of the block copolymer n‐PBDF‐*b*‐PBDT decreases from 2075 S cm^−1^ of n‐PBDF to 436 S cm^−1^ after the BDT monomers were polymerized. The decrease in conductivity is due to the low conductivity of the n‐PBDT polymer itself.^[^ ^16^, ^22^ ^]^ For comparison, the mixture of n‐PBDF and n‐PBDT was also prepared in a 1:1 volume ratio. The hydrodynamic diameter of the mixture is between n‐PBDF and n‐PBDT, lower than the block copolymer n‐PBDF‐*b*‐PBDT. These results demonstate the successful preparation of block copolymer n‐PBDF‐*b*‐PBDT. By adopting the opposite monomer addition sequence, i.e., adding the BDF monomer to n‐PBDT prepolymer solution, the block copolymer, n‐PBDT‐*b*‐PBDF, can also be obtained (Figure 5d, and see details in the Supporting Information).

## Conclusion

In summary, we developed an efficient, cost‐effective, and scalable MoO_3_ catalyzed polymerization of n‐PBDF, in which ppm levels of MoO_3_ lead to high monomer conversions (>99% by NMR), thus eliminating the need for any purification process. Importantly, through detailed mechanistic studies, we reveal that this catalytic polymerization proceeds via a MoO_3_ mediated DMSO oxidative pathway, in which DMS is detected as the reduction product. We also demonstrate that MoO_3_ catalyzed polymerization of n‐PBDF exhibits controlled chain‐growth polymerization characteristics, enabling the successful synthesis of block copolymers by the sequential addition of monomers. This new catalytic approach not only provides a cost‐effective, scalable route for synthesizing high‐quality n‐PBDF, greatly facilitating its mass production, commercialization, and widespread application across various fields, but also enriches our toolbox for polymer synthesis and enables the development of an innovative class of conducting block copolymers.

## Conflict of Interests

The authors declare the following competing financial interest(s): Patent disclosure has been filed by Purdue Research Foundation. J.M. is a co‐founder of Ambilight Inc., which partially sponsors the study under a research agreement. J.M. is a founder of PBDF LLC, which focuses on the commercialization of n‐PBDF.

## Supporting information



Supporting Information

## Data Availability

The data that support the findings of this study are available in the Supporting Information of this article.
