# Supplementary material for: Catalytic Polymerization of n‐Doped Poly(benzodifurandione) (n‐PBDF) Using Parts Per Million (ppm) Levels of Molybdenum Trioxide
Source: Angew Chem Int Ed Engl. 2025 Jul 11;64(36):e202510411. doi: 10.1002/anie.202510411 (PMC12402895; doi:10.1002/anie.202510411)
Supplement: Supplementary file 1 — Supporting Information [file ANIE-64-e202510411-s001.docx]

**Supporting Information**

**Catalytic Polymerization of n-Doped Poly(benzodifurandione) (n-PBDF) Using Parts Per Million (ppm) Levels of Molybdenum Trioxide**

*Guangchao Liu^1^, Uttam Pal^1^, Sanket Samal^1^, Michael F. Espenship^1^, Yuanhe Li^1^, Won-June Lee^1^, Lawal Adewale Ogunfowora^1^, Liyan You^1^, Julia Laskin^1^, Jianguo Mei^1^**

^1^James Tarpo Jr. and Margaret Tarpo Department of Chemistry, Purdue University, West Lafayette, Indiana 47907, United States

This file includes

1. General procedures and experimental details
2. Metal oxides catalyzed polymerization of n-PBDF
3. Polymerization conditions optimization of MoO_3_ catalyzed polymerization
4. Polymerization kinetics study of MoO_3_ catalyzed polymerization
5. Mechanistic understanding of MoO_3_ catalyzed polymerization
6. Scale-up synthesis of n-PBDF ink by using ppm level MoO_3_
7. Generalization and application of MoO_3_ catalyzed polymerization

References

1. **General procedures and experimental details**

**Materials**. All reagents and solvents for the synthesis and analysis were purchased from Sigma-Aldrich, Alfa Aesar, Thermo Fisher, and TCI and were used without further purification, unless otherwise mentioned. 3,7-dihydrobenzo[1,2-b:4,5-b′]difuran-2,6-dione (BDF) monomer was synthesized according to previous report.^[1]^ 3,7-dihydrobenzo[1,2-b:4,5-b']dithiophene-2,6-dione (BDT) monomer was synthesized according to previous report.^[2]^ All air and water sensitive reactions were performed under a nitrogen atmosphere.

**Characterizations**. ^1^H NMR spectra were recorded on Bruker ARX-400 (400 MHz). All chemical shifts were reported in parts per million (ppm). ^1^H NMR chemical shifts were referenced to DMSO-*d*_6_ (2.50 ppm). High resolution mass spectra (HRMS) were recorded on an Agilent 6560 Ion Mobility Quadrupole Time-of-Flight mass spectrometer. Ultraviolet–visible–near infrared (UV-Vis-NIR) absorption spectra were measured on an Agilent Cary 5000 UV–Vis spectrometer. Dynamic Light Scattering (DLS) was measured on Wyatt DynaPro NanoStar II instrument. The sheet resistance for drop-cast films of n-PBDF inks and conductivity mapping of the large-area transparent n-PBDF conductive thin film were measured by Filmetrics R50 Resistance Mapper. Film thickness was measured by Bruker DektakXT profilometer. The transmittance of the large-area transparent n-PBDF conductive film was measured by Filmetrics F10-RT thin film analyzer.

**Substrate and Film Preparation**. Glass substrate (19 × 19 mm) was cleaned by consecutive sonication in soap, DI water, acetone, and isopropanol for 15 min each. After drying the substrates with nitrogen, the substrates were exposed to UV-Ozone treatment (HELIOS-500 Ultraviolet-Ozone Cleaner) for 20 min to remove any organic residues and to increase surface hydrophilicity. Polymer thin films were coated on the glass substrates. Before drop casting, all the polymer inks were treated by a vortex mixer (Fisher Scientific) for 5 min. The desired thicknesses are around 1~2 nm. After coating, the film was dried in a vacuum oven (at 500 microns pressure at room temperature) to remove residual solvent. The large-area transparent organic conductor (TOC) film was prepared according to our previous work.^[3]^

**Conductivity and Sheet Resistance Measurements.** Drop-cast samples were prepared for polymer electrical conductivity measurements. The *R*_s_ (sheet resistance) of polymer films were obtained through the top-contact four-point probe (4PP) measurement by the Filmetrics R50 Resistance Mapper.

The conductivity of the films was then calculated by the equation below:

$$\sigma=\frac{1}{R_{s}t}$$

where *t* (film thickness) was measured by a profilometer.

**High-resolution Electrospray Ionization Mass Spectrometry (ESI-MS) Analysis**

Mass spectra were acquired on an Agilent 6560 Ion Mobility Quadrupole Time-of-Flight mass spectrometer (Santa Clara, CA, USA) equipped with a custom electrospray ionization (ESI) extension inlet heated to 300 °C with an applied 3.5 kV spray voltage. All spectra were acquired in negative ESI mode. A 1 mM sample solution of starting reagent in acetonitrile:DMSO (80:20 v:v) was infused directly into the inlet through a fused-silica capillary (50 μm ID, 150 μm OD) at a 1 μL/min flow rate using as syringe pump. The mass resolution (m/Δm) at *m/z* 379 was 21,000. Collision-induced dissociation (CID) was performed for select precursor ions by filtering through the instrument’s quadrupole mass filter before injection into a collision cell will background nitrogen gas at a collision energy of 10 eV before acquisition of a product ion scan.

1. **Metal oxides catalyzed polymerization of n-PBDF**


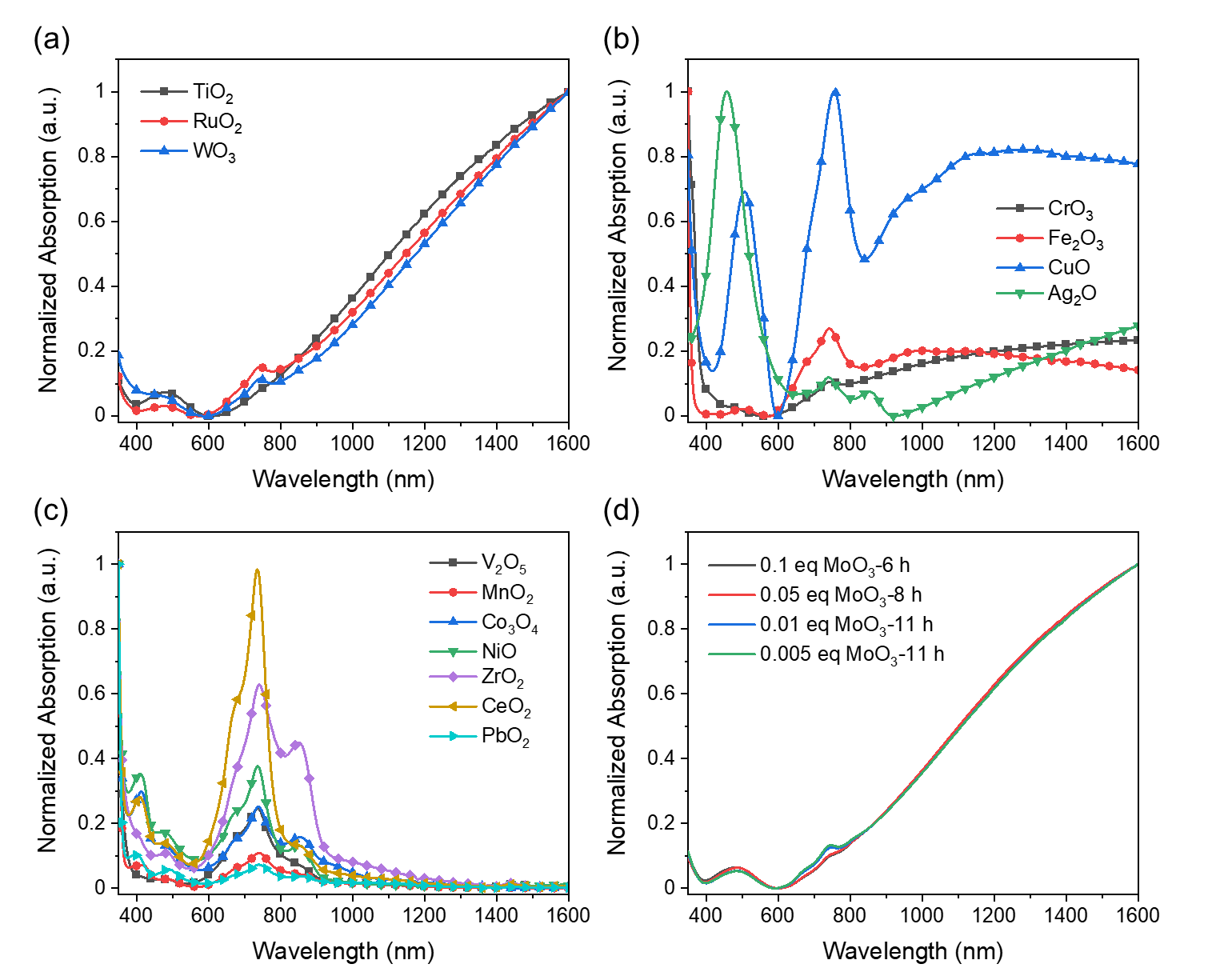


**Figure S1** UV-Vis-NIR absorption spectroscopy of polymerization mixtures obtained from (a), (b), (c) different metal oxides (polymerization conditions: 15 mg mL^−1^ BDF with 0.1 equiv. metal oxides at 100 °C for 24 h), and (d) MoO_3_ (polymerization conditions: 15 mg mL^−1^ BDF with different loadings of MoO_3_ at 100 °C).

**Table S1** Investigation of catalytic polymerization of n-PBDF by using different metal oxides.

| Entry | Metal oxides | Appearance | Conductivity (S cm^−1^) |
| --- | --- | --- | --- |
| 1 | TiO_2_ | Viscous solution | 1519 ± 42 |
| 2 | MoO_3_ | Insoluble gel | —*^a^* |
| 3 | RuO_2_ | Dilute Solution | 89 ± 12 |
| 4 | WO_3_ | Solution | 114 ± 14 |
| 5 | CrO_3_ | Dilute solution | NA*^b^* |
| 6 | Fe_2_O_3_ | Dilute solution | NA |
| 7 | CuO | Dilute solution | NA |
| 8 | Ag_2_O | Dilute solution | NA |
| 9 | V_2_O_5_ | Dilute solution | NA |
| 10 | MnO_2_ | Dilute solution | NA |
| 11 | Co_3_O_4_ | Dilute solution | NA |
| 12 | NiO | Dilute Solution | NA |
| 13 | ZrO_2_ | Dilute Solution | NA |
| 14 | CeO_2_ | Dilute Solution | NA |
| 15 | PbO_2_ | Dilute Solution | NA |

*^a^*Not obtained due to insoluble gel obtained in MoO_3_ mediated reaction. *^b^*Not obtained due to weak or no polaron and bipolar absorption in the NIR region of the mixtures obtained in other metal oxides mediated reactions. All reactions were conducted by using 0.1 equiv. meta oxides with 15 mg mL^−1^ BDF at 100 °C for 24 hours. All conductivities were obtained from dialyzed inks and measured by four-point probe method.

**Table S2** Investigation of catalytic polymerization by using MoO_3_ solid as catalyst.

| Entry | MoO_3_ (eqiuv.) | Time (h)*^a^* | Appearance | Conductivity (S cm^−1^) |
| --- | --- | --- | --- | --- |
| 1 | 0.1 | 24 | Insoluble gel | NA*^b^* |
| 2 | 0.1 | 6 | Viscous solution | 2483 ± 78 |
| 3 | 0.05 | 8 | Viscous solution | 2345 ± 65 |
| 4 | 0.01 | 11 | Viscous solution | 2445 ± 36 |
| 5 | 0.005 | 11 | Viscous solution | 2360 ± 53 |

*^a^*Since an insoluble gel was obtained in the polymerization catalyzed by 0.1 eq MoO₃ after 24 hours, all other polymerizations were stopped before formed gels. *^b^*Not obtained. All reactions were conducted by using MoO_3_ with 15 mg mL^−1^ BDF at 100 °C. All inks were purified by dialysis. All conductivities were obtained by four-point probe method.

1. **Polymerization conditions optimization of MoO_3_ catalyzed polymerization**

To explore the potential of catalytic polymerization at even lower catalyst loading, MoO_3_ and its hydrate molybdic acid (H_2_MoO_4_) were dissolved in DMSO to prepare their solutions (0.1 mg mL^−1^) before polymerization. MoO_3_ dissolves slowly in DMSO at around 140 ℃ to give a solution,^[4]^ and this process may take several days, while H_2_MoO_4_ can dissolve relatively easily at 100~140 ℃ in several hours. To optimize polymerization conditions and monitor polymerization process, time-dependent ^1^H-NMR spectra were recorded during the polymerization process. To quantitatively identify the monomer conversion and understand the degree of polymerization, an inert compound 1,2,4,5-Tetramethylbenzene (TMB) that does not affect polymerization was added as a standard. The conversions of BDF monomer were calculated from NMR integral using TMB as reference.

General procedure for ^1^H NMR characterization of polymerizations

The BDF monomer (from 5 mg mL^−1^ to 15 mg mL^−1^) and 1,2,4,5-Tetramethylbenzene (1 equiv.) were fully dissolved in DMSO-*d*_6_ at various temperatures (from 80 ℃ to 160 ℃) under nitrogen atmosphere, then MoO_3_ solution (0.1 mg mL^−1^ DMSO-*d*_6_ solution, from 0.001 equiv. to 0.00001 equiv.) was added. The total volume of DMSO-*d*_6_ is 10 mL. The mixture continued stirring for several hours until the gels formed or the monomer conversion was complete. During which 0.5 mL of the mixture was charged into an NMR tube and ^1^H-NMR spectra were recorded at different polymerization intervals. The conversions of BDF monomer were calculated from NMR integral in aromatic region using TMB as reference. The results are shown in **Figures S2-S11**.

**
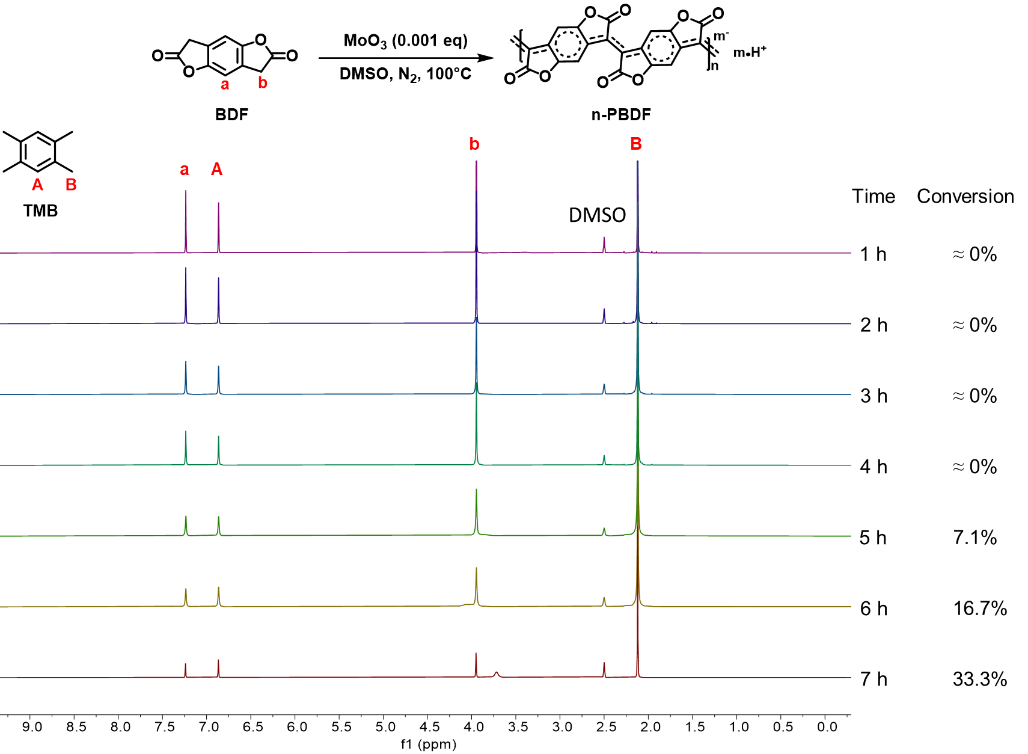
**

**Figure S2** ^1^H NMR spectrum of polymerization of BDF (15 mg mL^−1^) with MoO_3_ (0.001 eq) in DMSO-*d*_6_ at 100 ℃ for different time under nitrogen. TMB was used as a reference. The reaction mixture formed gel after 7 hours.


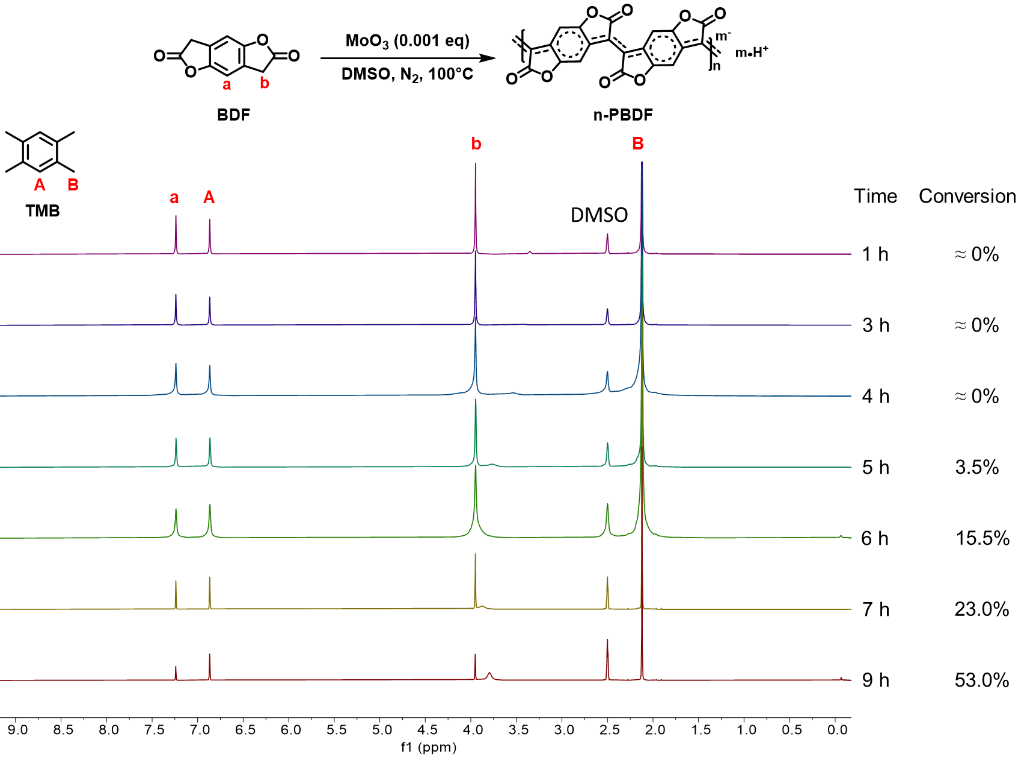


**Figure S3** ^1^H NMR spectrum of polymerization of BDF (10 mg mL^−1^) with MoO_3_ (0.001 eq) in DMSO-*d*_6_ at 100 ℃ for different time under nitrogen. TMB was used as a reference. The reaction mixture formed gel after 9 hours.


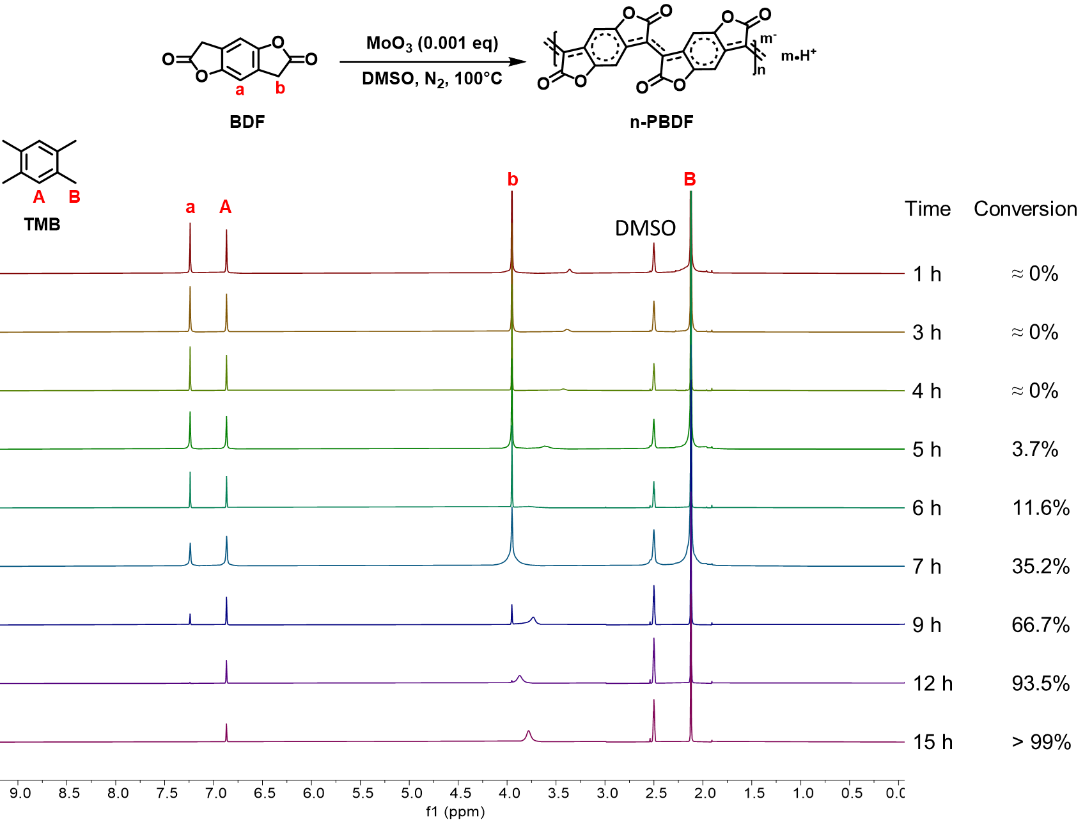


**Figure S4** ^1^H NMR spectrum of polymerization of BDF (7.5 mg mL^−1^) with MoO_3_ (0.001 eq) in DMSO-*d*_6_ at 100 ℃ for different time under nitrogen. TMB was used as a reference.


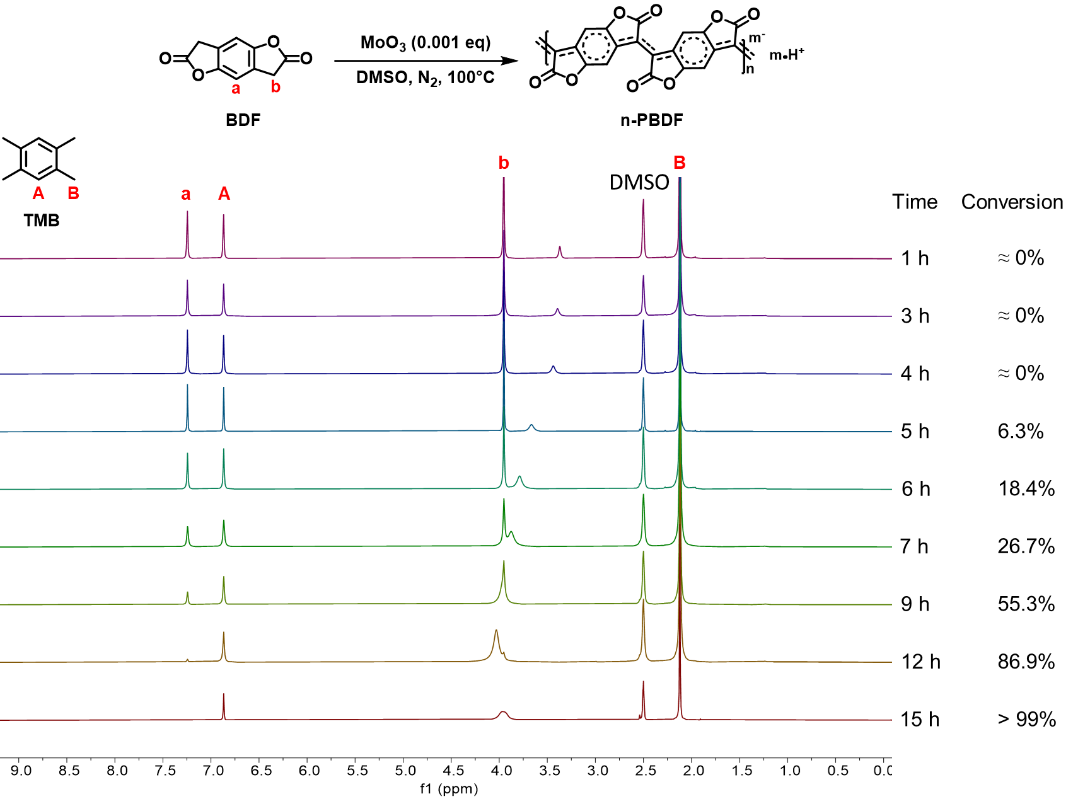


**Figure S5** ^1^H NMR spectrum of polymerization of BDF (5 mg mL^−1^) with MoO_3_ (0.001 eq) in DMSO-*d*_6_ at 100 ℃ for different time under nitrogen. TMB was used as a reference.


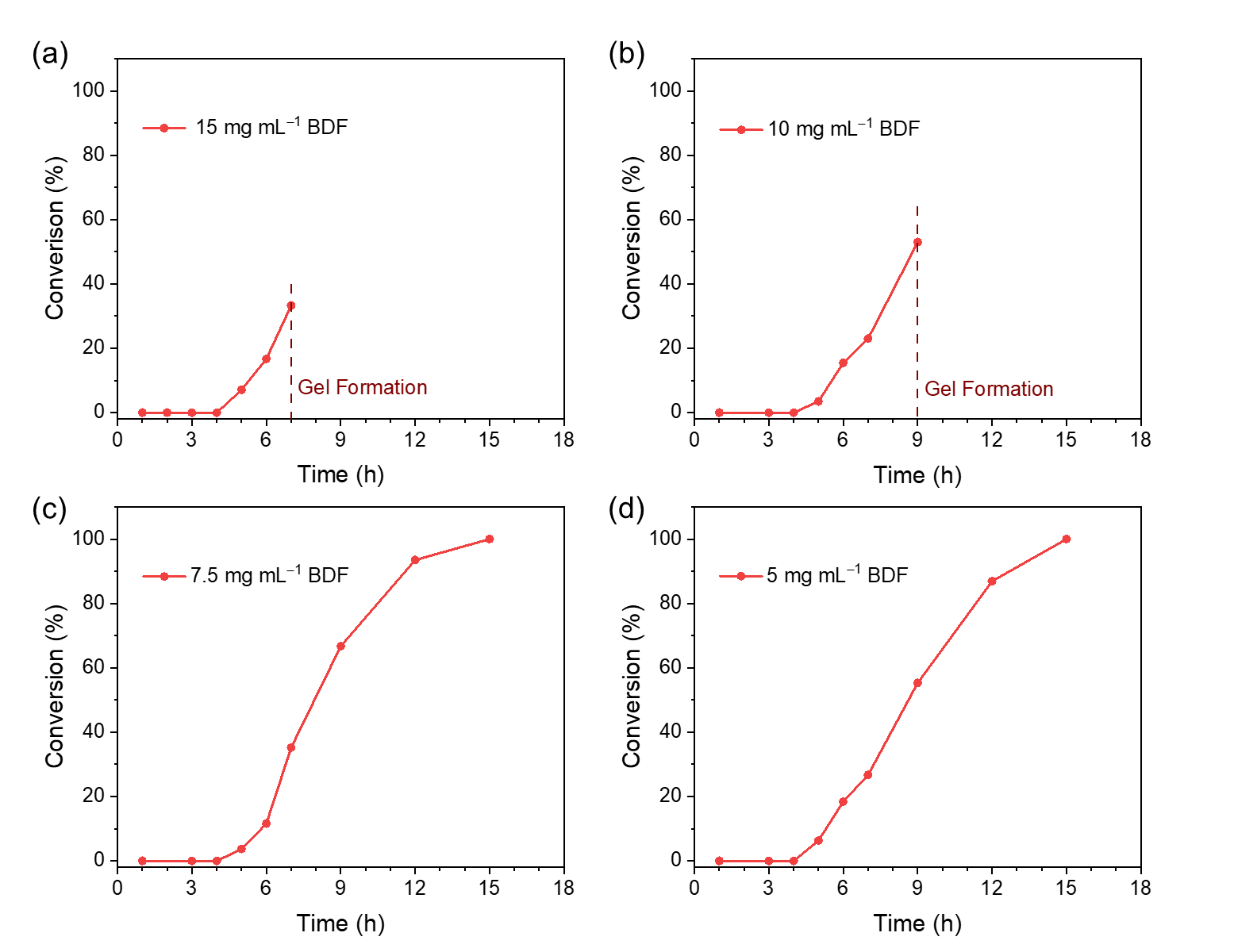


**Figure S6** Plots of monomer conversions with times under different monomer concentrations. (a) 15 mg mL^−1^. (b) 10 mg mL^−1^. (c) 7.5 mg mL^−1^. (d) 5 mg mL^−1^. (Polymerization conditions: BDF monomer with 0.001 equiv. MoO_3_ at 100 ℃).


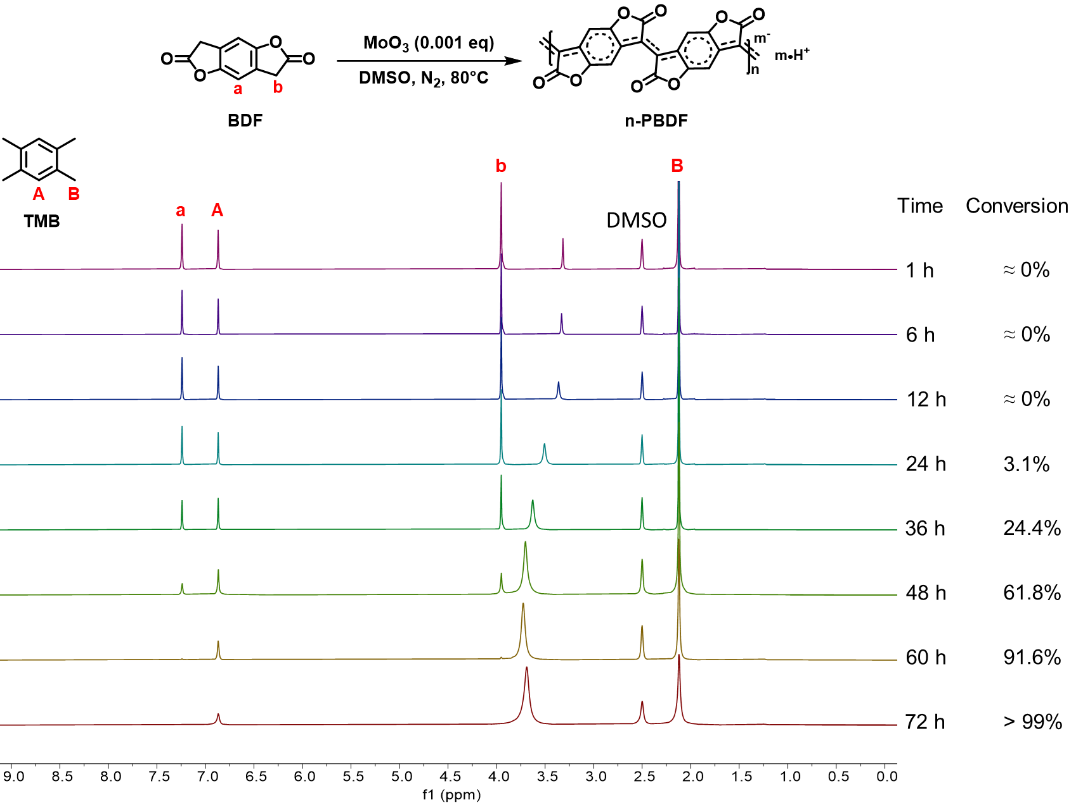


**Figure S7** ^1^H NMR spectrum of polymerization of BDF (5 mg mL^−1^) with MoO_3_ (0.001 eq) in DMSO-*d*_6_ at 80 ℃ for different time under nitrogen. TMB was used as a reference.


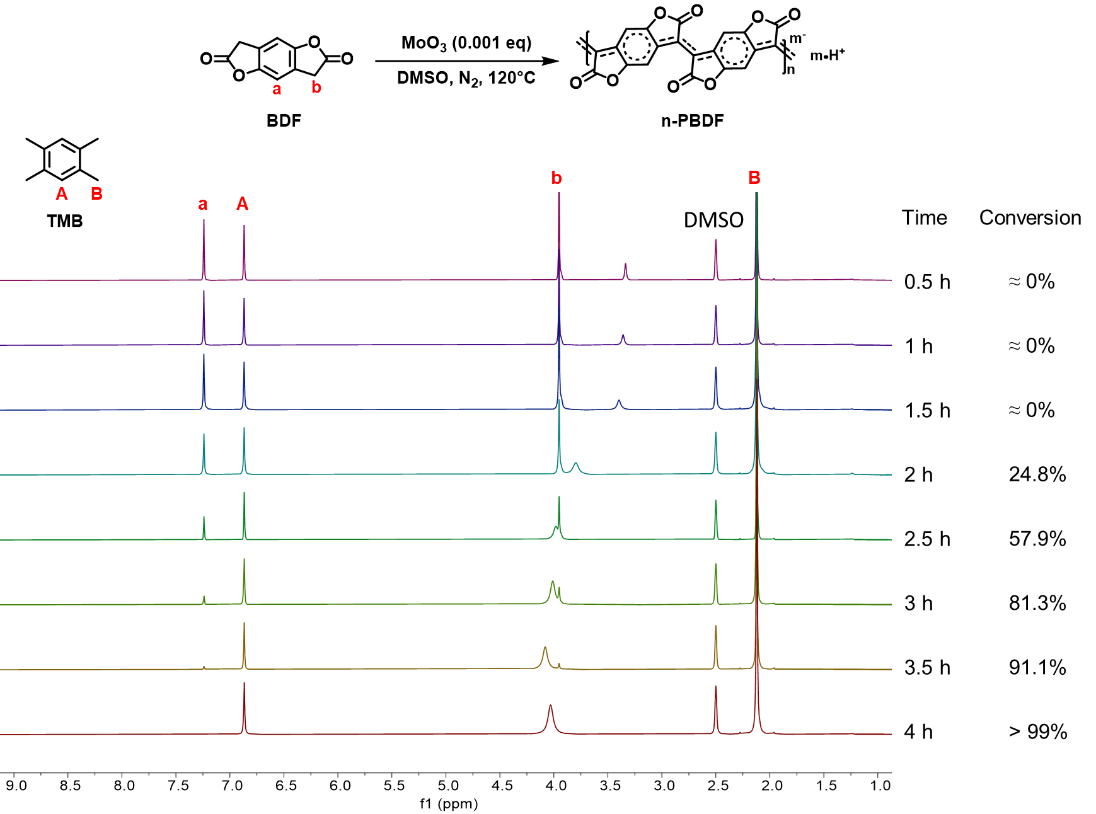


**Figure S8** ^1^H NMR spectrum of polymerization of BDF (5 mg mL^−1^) with MoO_3_ (0.001 eq) in DMSO-*d*_6_ at 120 ℃ for different time under nitrogen. TMB was used as a reference.


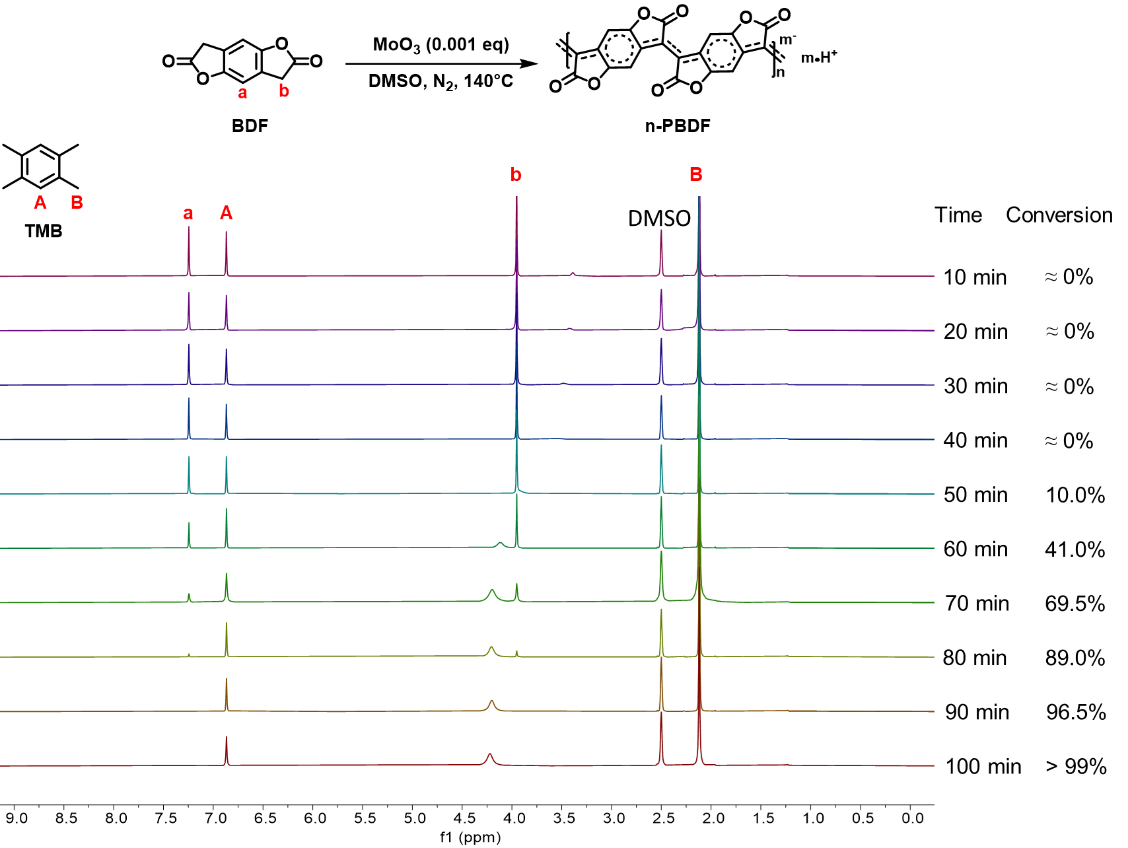


**Figure S9** ^1^H NMR spectrum of polymerization of BDF (5 mg mL^−1^) with MoO_3_ (0.001 eq) in DMSO-*d*_6_ at 140 ℃ for different time under nitrogen. TMB was used as a reference.


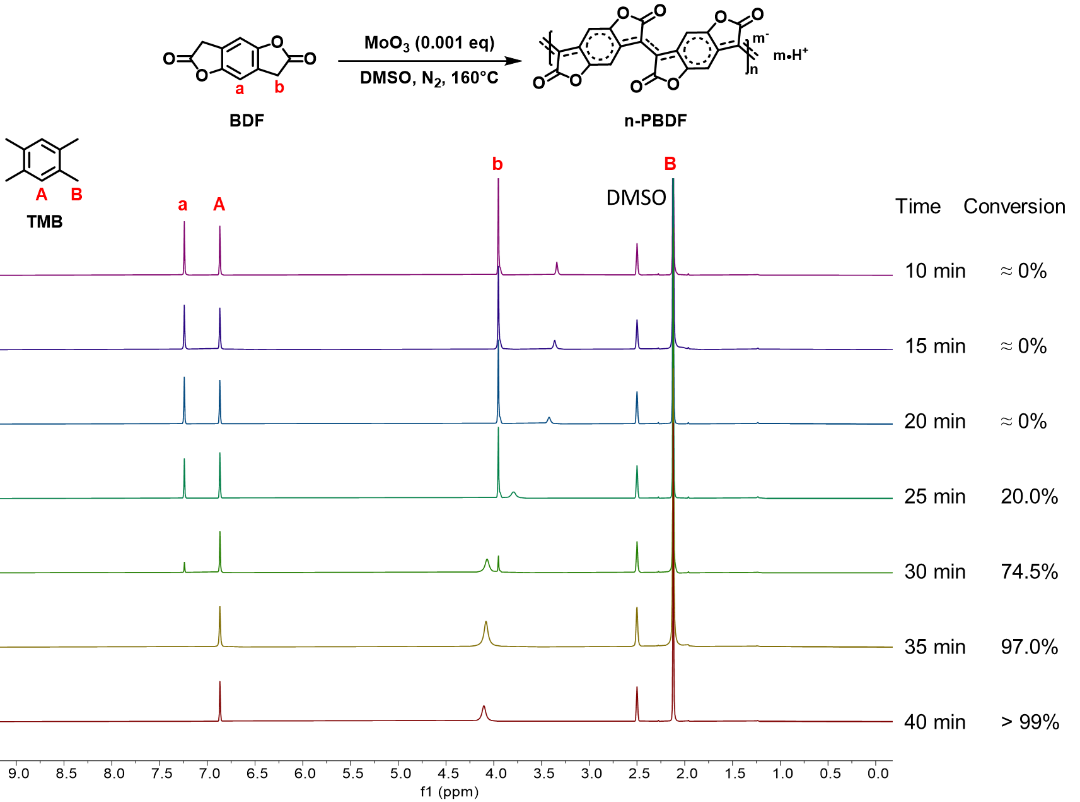


**Figure S10** ^1^H NMR spectrum of polymerization of BDF (5 mg mL^−1^) with MoO_3_ (0.001 eq) in DMSO-*d*_6_ at 160 ℃ for different time under nitrogen. TMB was used as a reference.


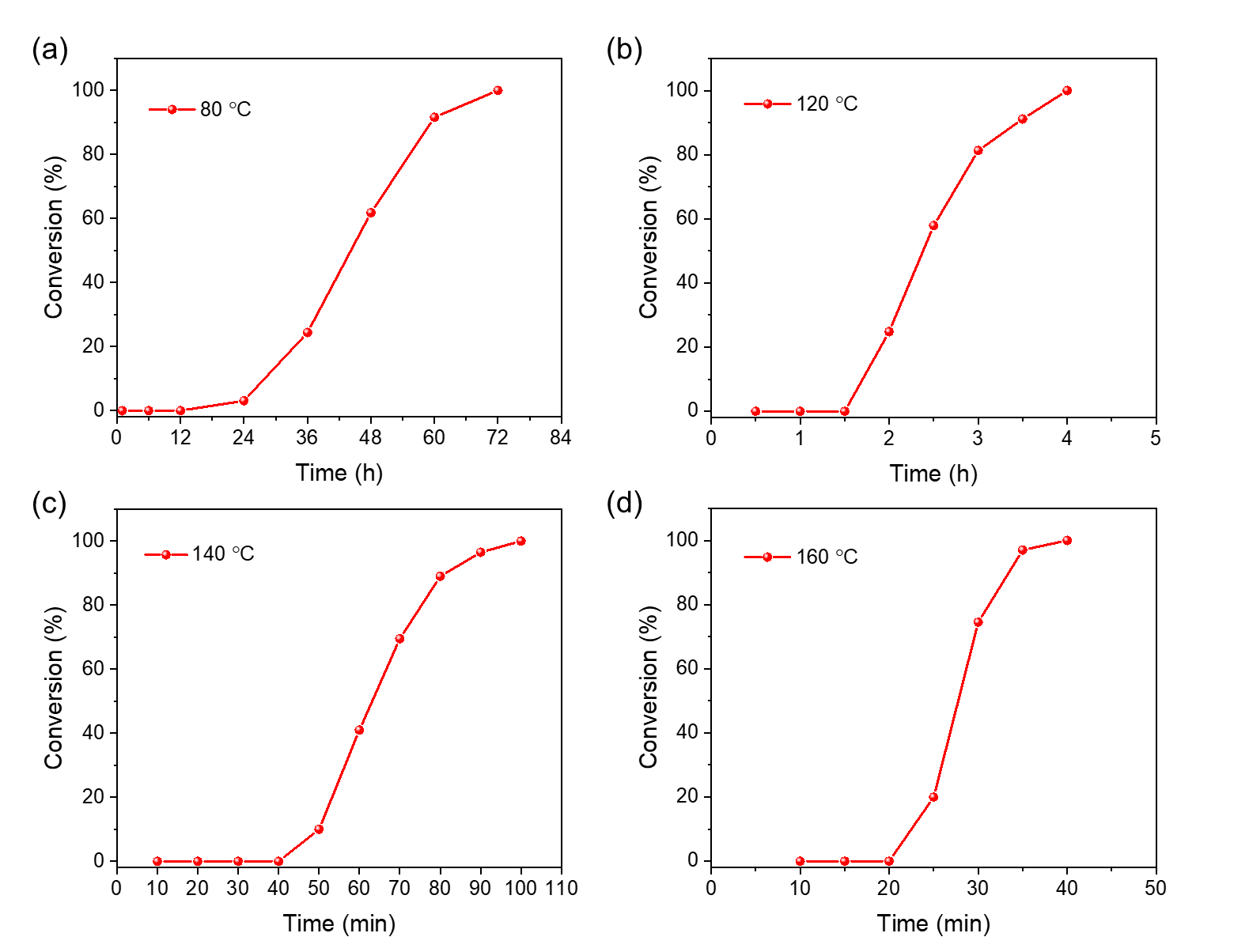


**Figure S11** Plots of monomer conversions with times at different temperatures. (a) 80 ℃. (b) 120 ℃. (c) 140 ℃. (d) 160 ℃. (Polymerization conditions: 5 mg mL^−1^ BDF monomer with 0.001 equiv. MoO_3_).

General procedure for the polymerization of n-PBDF: The BDF monomer (from 5 mg mL^−1^ to 15 mg mL^−1^) was dissolved in DMSO at various temperatures (from 80 ℃ to 160 ℃) under nitrogen atmosphere. Then MoO_3_ or H_2_MoO_4_ (0.1 mg mL^−1^ DMSO solution, from 0.001 equiv. to 0.00001 equiv.) was added into the reaction system. The total volume of DMSO is 10 mL. The mixture continued stirring for several hours until the gels formed, or the monomer conversion was complete (checked by ^1^H NMR). After the solution was cooled to room temperature, half-volume of the mixture was dialyzed against DMSO using a dialysis bag with a cut-off molecular weight of 10 kDa (Viskase, USA) to remove low molecular weight fractions. The electrical conductivities of the dialyzed ink and undialyzed ink were measured. The polymerization conditions and conductivity results were summarized in **Table 1** in the main text.

1. **Polymerization kinetics study of MoO_3_ catalyzed polymerization**

To further understand the polymerization kinetics and mechanisms, we try to obtain molecular weight information of the polymers. However, n-PBDF polymer is almost exclusively soluble in DMSO, and conventional GPC mobile phase is not applicable for its molecular weight measurement, such as tetrahydrofuran and chloroform. Therefore, we use Dynamic Light Scattering (DLS) measurements to characterize polymer hydrodynamic diameter and understand the polymerization kinetics. Although a qualitative relationship is difficult to determine for a doped polymer with possible aggregation behavior, it is clear that there they are positively correlated.

**Dynamic Light Scattering (DLS) measurements of n-PBDF polymers**

A general procedure for Dynamic Light Scattering (DLS) characterization of polymerization: The BDF monomer (50 mg, 5 mg mL^−1^) was dissolved in DMSO at various temperatures (100 ℃ or 140 ℃) under nitrogen atmosphere. Then MoO_3_ (DMSO solution, from 0.001 equiv. to 0.00001 equiv.) was added into the reaction system. The total volume of DMSO is 10 mL. The mixture continued stirring for several hours until the monomer conversion was complete (checked by ^1^H NMR). 0.02 mL of mixture was extracted from the reaction solution at different reaction times and diluted to 3 mL with DMSO. These dilutes solutions were used for time-dependent ultraviolet−visible−near infrared (UV‒Vis‒NIR) absorption measurements and Dynamic Light Scattering (DLS) studies. The DLS were summarized in **Tables S3-S5** and **Figures S12-S14**.

**Table S3** Hydrodynamic diameters of n-PBDF particles at different polymerization time (polymerization condition: 5 mg mL^−1^ BDF with 0.001 equiv. MoO_3_ at 100 ℃).

| Time | Monomer conversion (%)*^a^* | Hydrodynamic diameter (nm) |
| --- | --- | --- |
| 1 h | 0 | —*^b^* |
| 2 h | 0 | — |
| 3 h | 0 | — |
| 4 h | 0 | — |
| 5 h | 6.3 | — |
| 6 h | 18.4 | 26.7 ± 1.1 |
| 7 h | 26.7 | 42.5 ± 1.5 |
| 9 h | 55.3 | 58.4 ± 2.4 |
| 12 h | 86.9 | 67.5 ± 1.0 |
| 15 h | >99 | 71.7 ± 2.0 |
| 18 h | >99 | 72.3 ± 1.6 |
| 24 h | >99 | 72.8 ± 1.8 |

*^a^*Obtained from ^1^H NMR. *^b^*No detectable hydrodynamic diameter data.


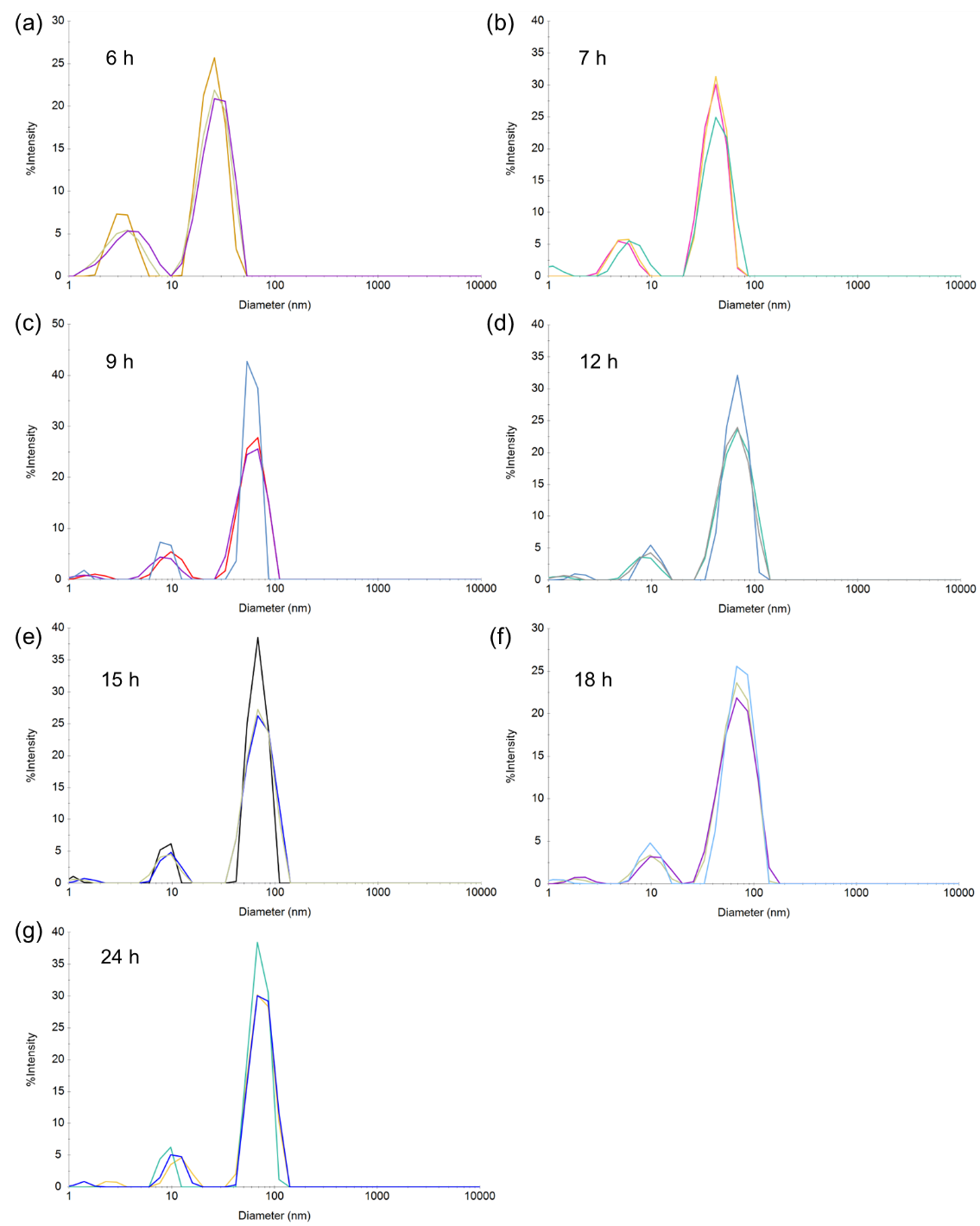


**Figure S12** Dynamic light scattering (DLS) analysis of hydrodynamic diameter distribution of n-PBDF particles at different polymerization time (polymerization condition: 5 mg mL^−1^ BDF with 0.001 equiv. MoO_3_ at 100 ℃).

**Table S4** Hydrodynamic diameters of n-PBDF particles at different polymerization time (polymerization condition: 5 mg mL^−1^ BDF with 0.001 equiv. MoO_3_ at 140 ℃).

| Time | Monomer conversion (%)*^a^* | Hydrodynamic diameter (nm) |
| --- | --- | --- |
| 10 min | 0 | —*^b^* |
| 20 min | 0 | — |
| 30 min | 0 | — |
| 40 min | 0 | — |
| 50 min | 10.0 | 24.0 ± 1.5 |
| 60 min | 41.0 | 40.3 ± 1.0 |
| 70 min | 69.5 | 45.5 ± 1.1 |
| 80 min | 89 | 49.5 ± 1.5 |
| 90 min | 96.5 | 50.4 ± 1.7 |
| 100 min | >99 | 51.4 ± 0.8 |
| 2 h | >99 | 51.9 ± 1.9 |
| 3 h | >99 | 51.6 ± 0.8 |
| 6 h | >99 | 51.4 ± 1.7 |
| 12 h | >99 | 50.7 ± 1.7 |
| 24 h | >99 | 50.9 ± 1.0 |

*^a^*Obtained from ^1^H NMR. *^b^*No detectable hydrodynamic diameter data.


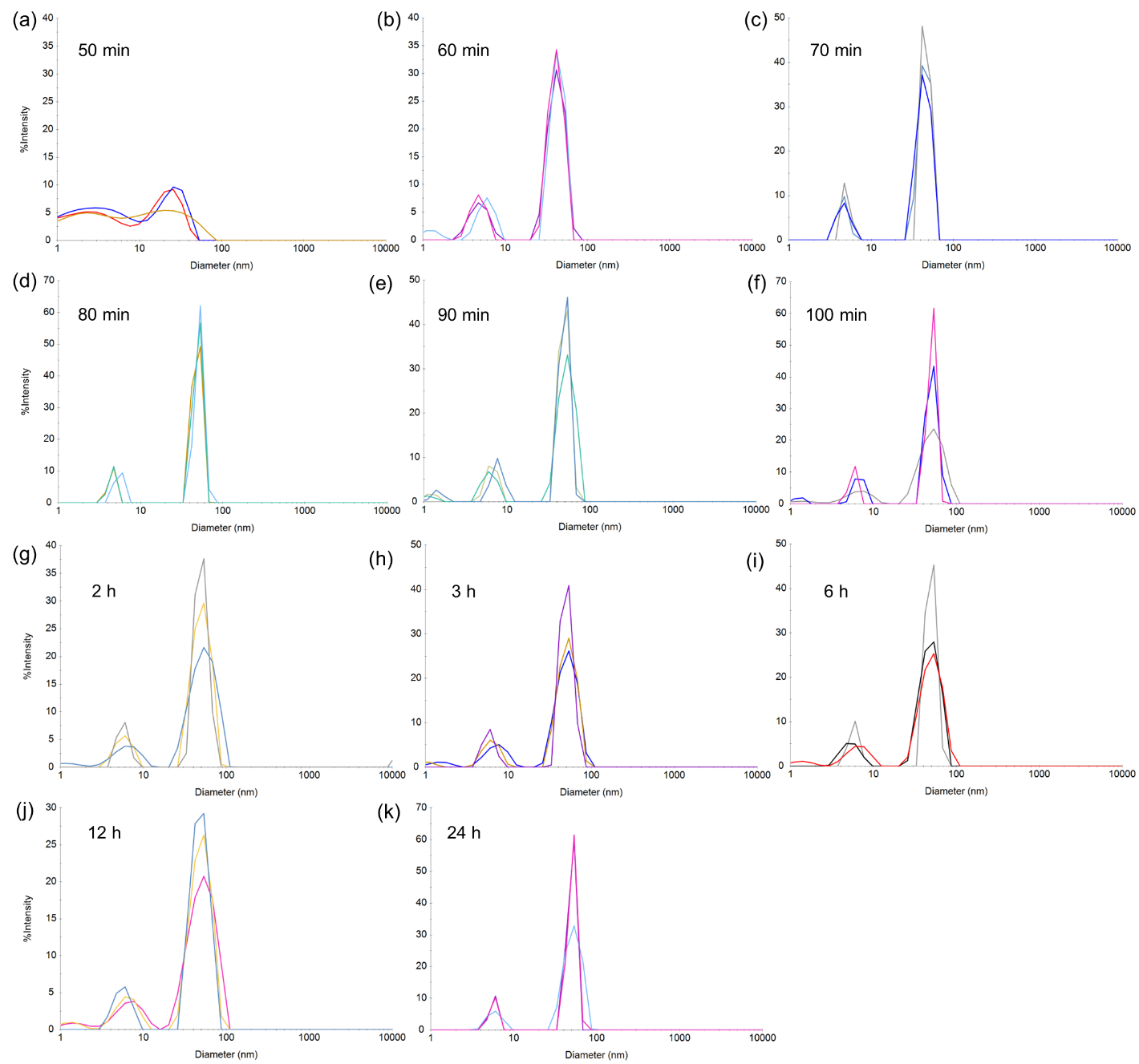


**Figure S13** Dynamic light scattering (DLS) analysis of hydrodynamic diameter distribution of n-PBDF particles at different polymerization time (polymerization condition: 5 mg mL^−1^ BDF with 0.001 equiv. MoO_3_ at 140 ℃).

**Table S5** Investigation of MoO_3_ catalyzed polymerization under different [BDF]/[MoO_3_] feed ratio at 140 ℃. (BDF was 5 mg mL^−1^).

| MoO_3_ loading (eq) | [BDF]/[MoO_3_] | Polymerization time (h)*^a^* | Appearance | Monomer conversion (%)*^b^* | Hydrodynamic diameter (nm)*^c^* |
| --- | --- | --- | --- | --- | --- |
| 0.001 | 1000 | 1.67 | Solution | >99 | 51.4 ± 0.8 |
| 0.0005 | 2000 | 2.5 | Solution | >99 | 54.2 ± 1.0 |
| 0.0001 | 10000 | 6 | Solution | >99 | 60.0 ± 2.6 |
| 0.00005 | 20000 | 9 | Solution | >99 | 62.6 ± 1.4 |
| 0.00002 | 50000 | 12 | Solution | >99 | 73.2 ± 1.6 |
| 0.00001 | 100000 | 24 | Solution | >99 | 81.0 ± 1.0 |

*^a^*The reactions were stopped upon complete monomer conversion. *^b^*Complete monomer conversion checked by ^1^H NMR. *^c^*Measured from dynamic light scattering (DLS).


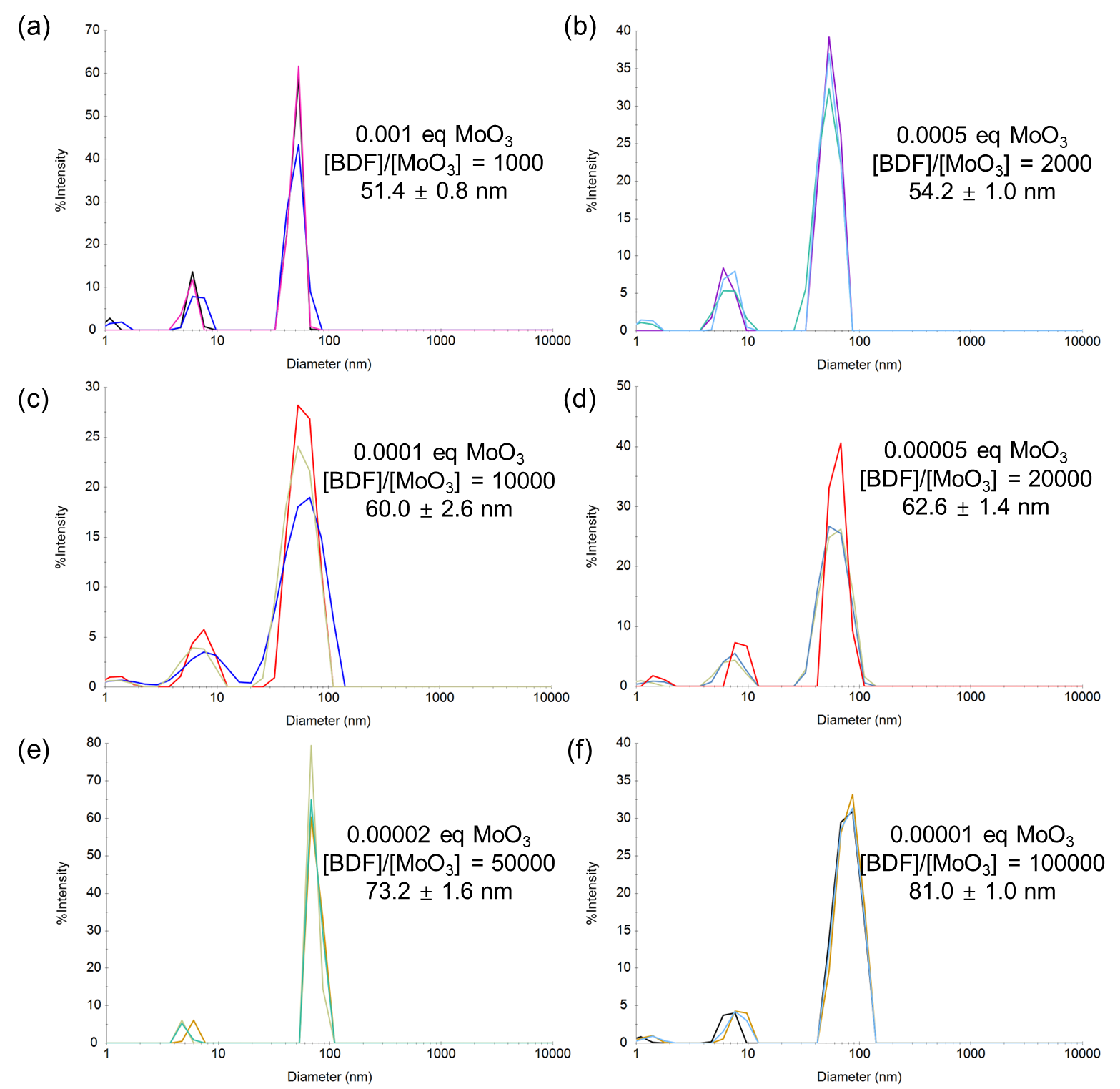


**Figure S14** DLS analysis of hydrodynamic diameter distribution of n-PBDF polymers under different [BDF]/[MoO_3_] feed ratio at 140 ℃ (BDF was 5 mg mL^−1^). (a) [BDF]/[MoO_3_] = 1000. (b) [BDF]/[MoO_3_] = 2000. (c) [BDF]/[MoO_3_] = 10000. (d) [BDF]/[MoO_3_] = 20000. (e) [BDF]/[MoO_3_] = 50000. (f) [BDF]/[MoO_3_] = 100000.

**“Monomer-addition” experiment and control experiments**

To have a deeper understanding of the polymerization kinetics, the chain-growth nature of the polymerization was examined by a “monomer-addition” experiment. The “monomer-addition” experiment and control experiments were described as follow:

1. Prepolymer synthesis: 5 mg mL^−1^ BDF monomer and 0.001 eq MoO_3_ (DMSO solution) were stirring at 100 ℃ under N_2_ atmosphere (10 mL reaction) until the monomer conversion was complete after 15 hours (checked by ^1^H NMR).
2. “Monomer-addition” experiment: At 100 ℃ under N_2_ atmosphere, a fresh batch of BDF monomer (DMSO solution, 5 mg mL^−1^, 2 mL) was added into 2 mL of the above-mentioned prepolymer solution, the mixture continued to polymerize for another 6 hours until the monomer conversion was complete (checked by ^1^H NMR).
3. The first control experiment: At 100 ℃ under N_2_ atmosphere, a fresh batch of BDF monomer (DMSO solution, 5 mg mL^−1^, 2 mL) and 0.001 eq MoO_3_ (DMSO solution) were added into 2 mL of the above-mentioned prepolymer solution, the mixture continued to polymerize for another 6 hours until the monomer conversion was complete (checked by ^1^H NMR).
4. The second control experiment: At 100 ℃ under N_2_ atmosphere, 2 mL of the above-mentioned polymer solution continued stirring for 6 hours.
5. The third control experiment: At 100 ℃ under N_2_ atmosphere, 2 mL DMSO was added into the above-mentioned prepolymer solution, the mixture continued stirring for 6 hours.

The prepolymer and the polymers from “monomer-addition” experiment and three control experiments were characterized by DLS to monitor the change of polymer particles sizes. The results were summarized in **Figure 2f** in the main text and **Figure S15**.


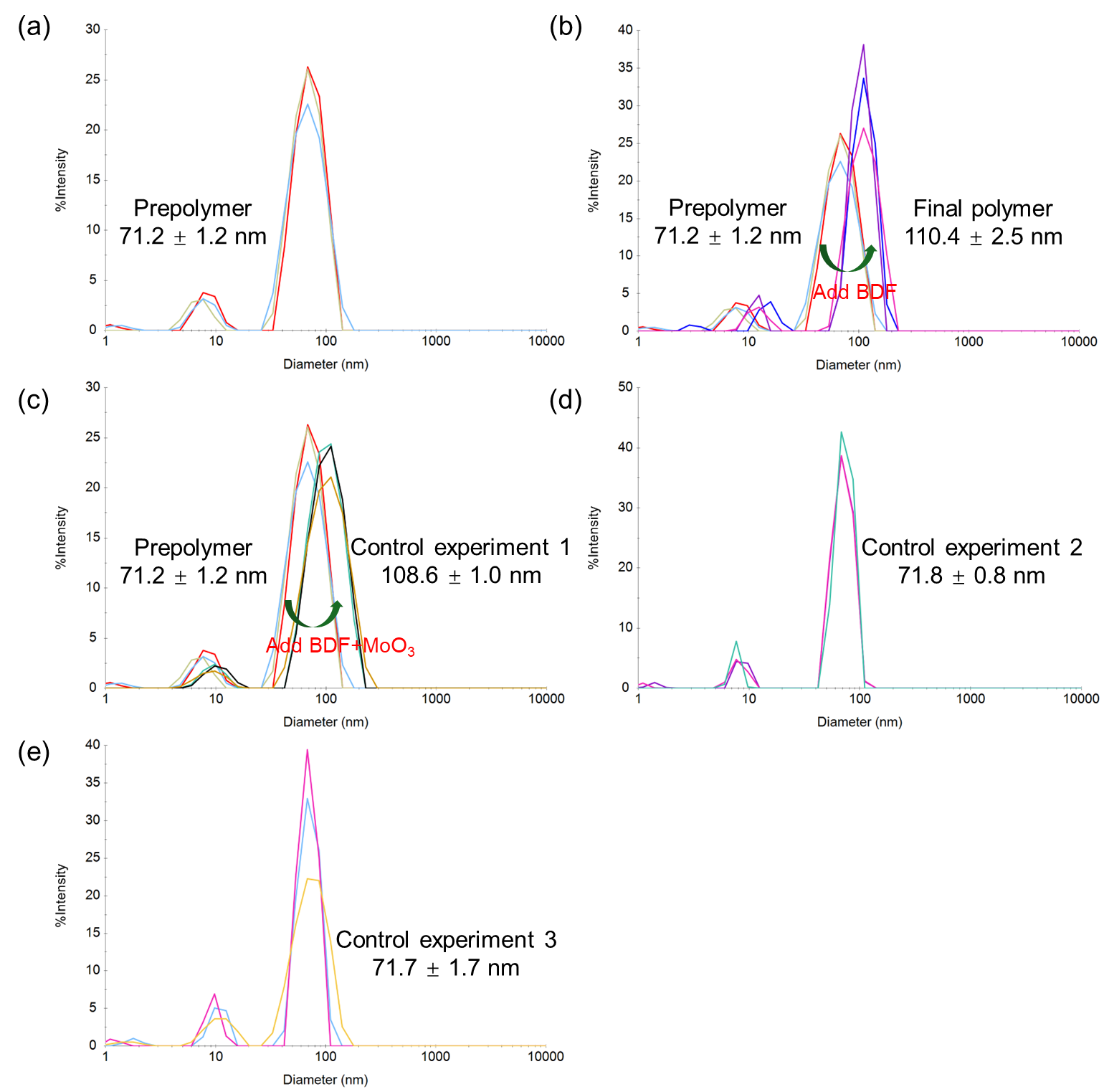


**Figure S15** DLS analysis of hydrodynamic diameter distribution of the prepolymer and the polymers from “monomer-addition” experiment and three control experiments. (a) DLS of prepolymer. (b) DLS of final polymer from “monomer-addition” experiment. The stacked DLS curve shows a clear increase in hydrodynamic diameter. (c) DLS from the first control experiment. The stacked DLS curve shows a clear increase in hydrodynamic diameter. (d) DLS from the second control experiment. (e) DLS from the third control experiment.

1. **Mechanistic understanding of MoO_3_ catalyzed polymerization**

To determine whether MoO_3_ also functions as an oxidant in the oxidative polymerization mechanism, we attempted to analyze the reaction orders of BDF and MoO_3_, which could provide critical insights into the reaction pathway. The detailed kinetic study was based on polymerization conducted in DMSO solvent, the ^1^H NMR spectrum of polymerization of BDF (5 mg mL^−1^) with MoO_3_ (0.001 eq) in DMSO at 140 ℃ were monitored, with the results summarized in **Figure S16**.

Our previous work has demonstrated that PBDF can be doped by H_2_O and BDF monomer.^[5]^ Given that DMS should have the stronger reducing ability than H_2_O, PBDF could also be doped by the DMS generated during the polymerization. The possible doping pathway of PBDF by DMS was proved by a series of re-doping experiments. The detailed results were shown in **Figure S17.**

To understand the mechanism of oxidative polymerization of n-PBDF, ESI-MS of the mixtures at the initial stage of the polymerizations were performed (**Figures S18-S19)**. The ESI-MS results show that in the early stage of polymerization, in addition to the BDF monomer, the diketone intermediate, dimer and the charge transfer complex (CTC) of two BDF were obviously detected.

To further investigate the polymerization mechanism, two polymerizations were conducted using 0.1 equivalents of H_2_MoO_4_, with and without adding 1 equivalent of *N*-*tert*-Butyl-α-phenylnitrone (PBN), a commonly used radical trapping agent. Using 0.1 equivalent of catalyst was intended to generate more possible radical intermediates for trapping by PBN and detection by ESI-MS. The ESI-MS spectra and results were summarized in **Figures S20-S21**. The ^1^H NMR and UV–Vis–NIR absorption spectra of the mixtures obtained from the two polymerizations were summarized in **Figure S22**.

The detailed proposed mechanism for the MoO_3_-DMSO complex mediated dimerization process was summarized in **Figure S23**. With the mechanistic understanding of activated-DMSO oxidation pathway, we further explored other DMSO activation reagents (eg. carbodiimide or acylating agents) in mediating the oxidative polymerization of BDF and proposed a general DMSO oxidation pathway of BDF monomers by using DMSO-activating agents (**Figures S24-S25**).


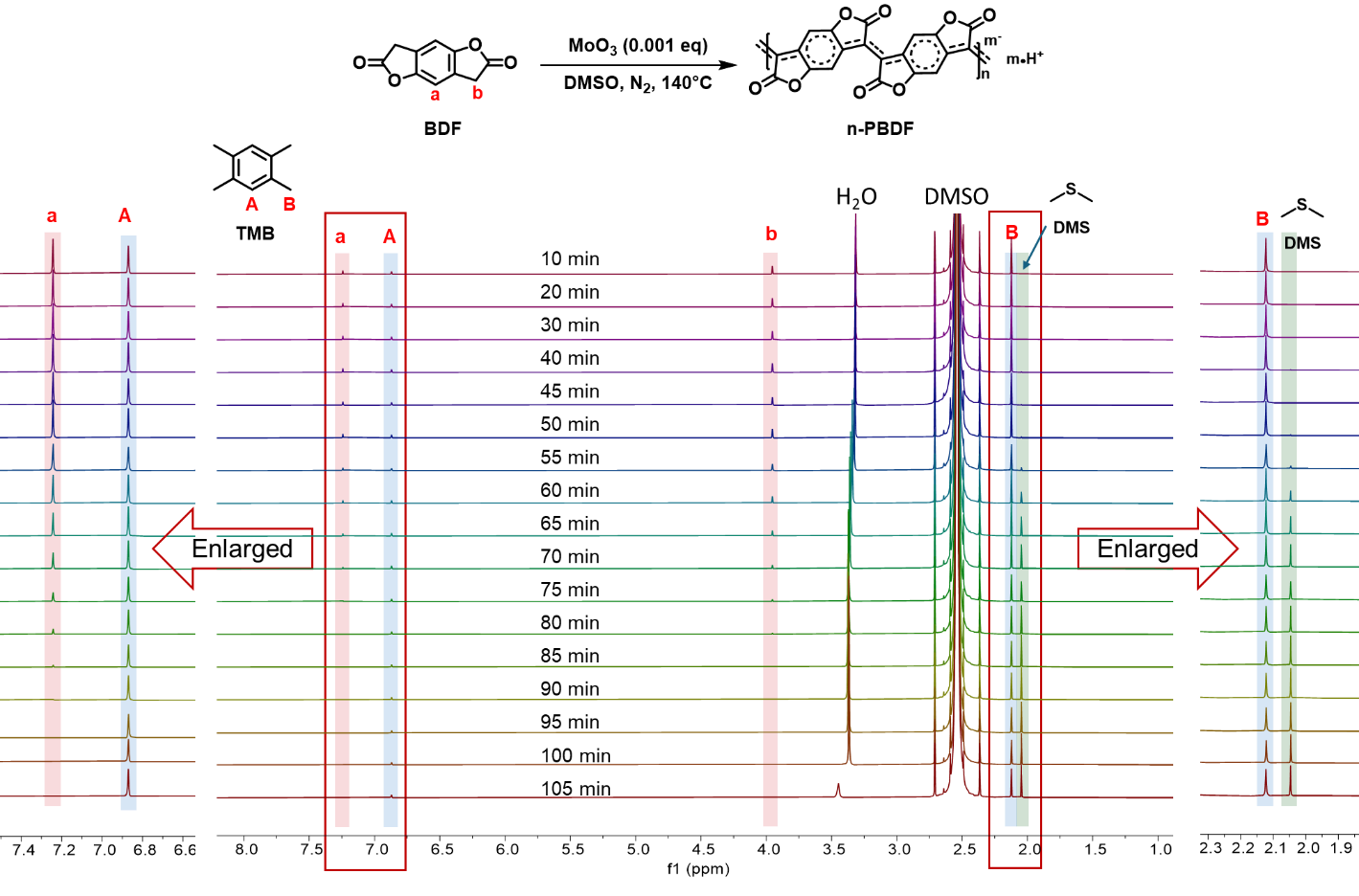


**Figure S16** ^1^H NMR spectrum of polymerization of BDF (5 mg mL^−1^) with MoO_3_ (0.001 eq) in DMSO at 140 ℃ for different time under nitrogen. TMB was used as a reference. A new peak gradually emerged at 2.05 ppm, which was assigned to DMS by comparing with the chemical shift values reported in the literature.^[6]^

The procedures for the dedoping of n-PBDF and re-doping of PBDF were performed according to our previous report.^[5c]^ n-PBDF thin films were prepared by off-cent spin coating method on the glass substrate (19 × 19 mm) and the thickness of n-PBDF thin film was controlled around 40~50 nm. To avoid the impact of water and oxygen, the dedopant (Magic Blue) solutions preparation and dedoping reactions were performed in the glove box. For the dedoping reaction, a sequential dedoping method was employed, which performed by dropping 200 μL acetonitrile solution of the Magic Blue (10 mM) to cover the whole surface of n-PBDF thin film and then the sample was quickly spin-cast at 2000 rpm for 1 min. Afterward, the film was rinsed with pure acetonitrile to remove excess dopants and byproducts. In the redoping reaction, dedoped PBDF films were immersed in the DMS solutions for different times. The residual solvent after the treatment can be dried by nitrogen gun, and placed under vacuum for 10 min. The UV-Vis-NIR absorption spectra of the as-cast n-PBDF, as-dedoped PBDF, and redoped PBDF thin films were measured, with the results summarized in **Figure S17**.


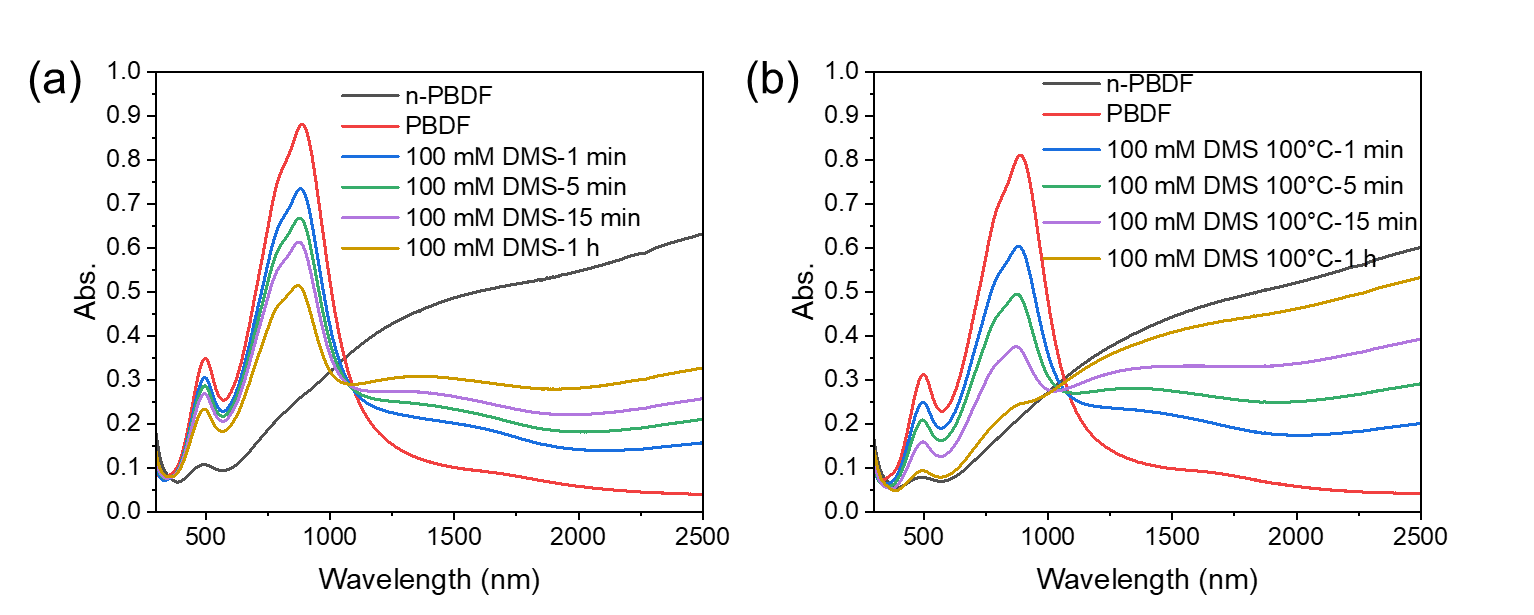


**Figure S17** The UV-Vis-NIR absorption of as-cast n-PBDF, as-dedoped PBDF, and redoped PBDF thin films treated with 100 mM DMS solution in DMSO at (a) RT and (b) 100 ℃ for different times. There is a gradual increase of polaron and bipolaron absorption bands in the NIR region and a decrease of strong neutral peaks at 895 and 500 nm when the PBDF thin film was immersed in DMS solution at RT, indicating the gradually redoping of PBDF by DMS. After the solution was heated to 100 ℃, PBDF thin films exhibited faster redoping speed, with almost fully recovered bipolaron and polaron absorption after treated for 1 hour at 100 ℃.

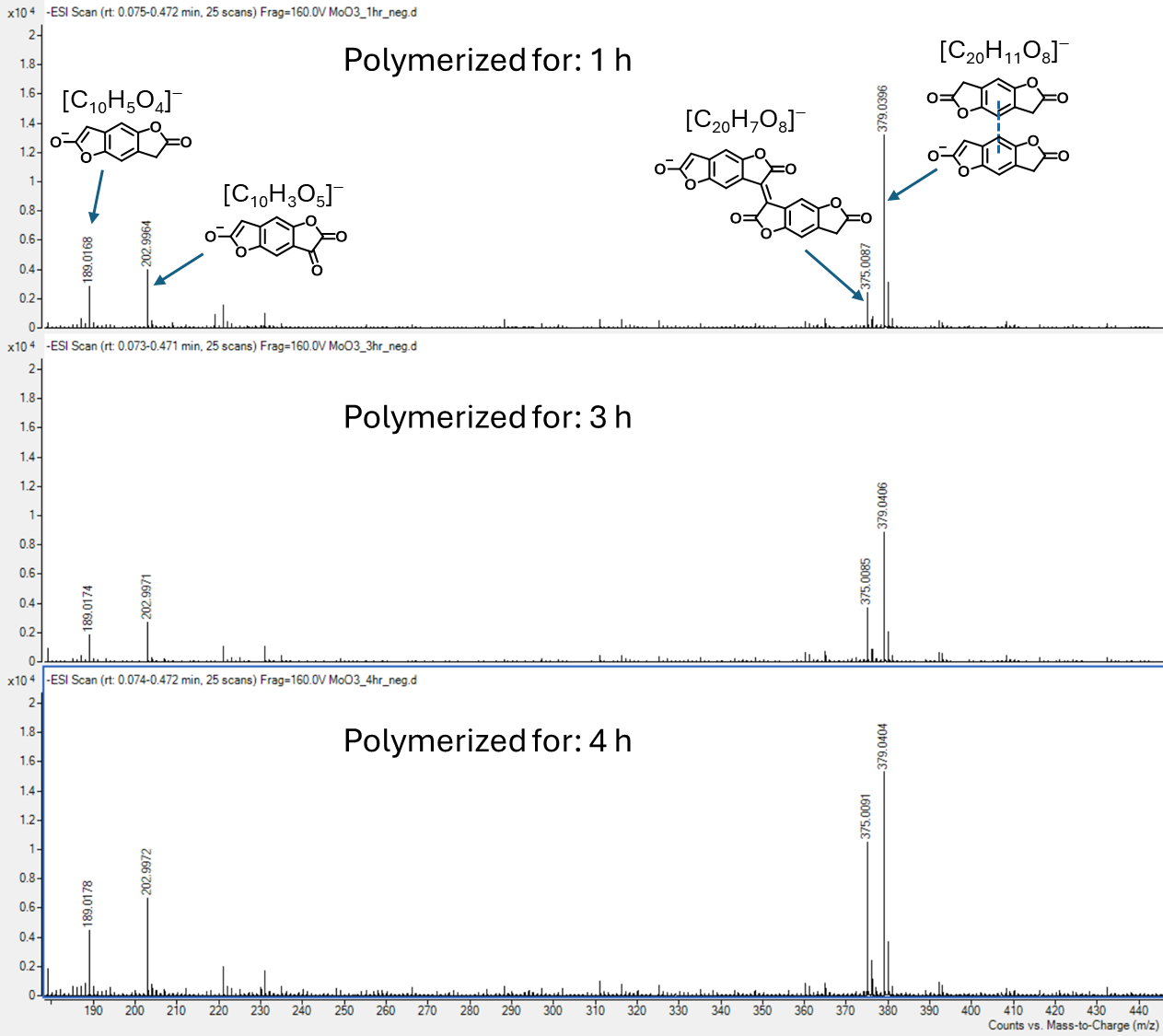


**Figure S18** ESI-MS of the mixtures from the polymerization with 0.001 eq MoO_3_.

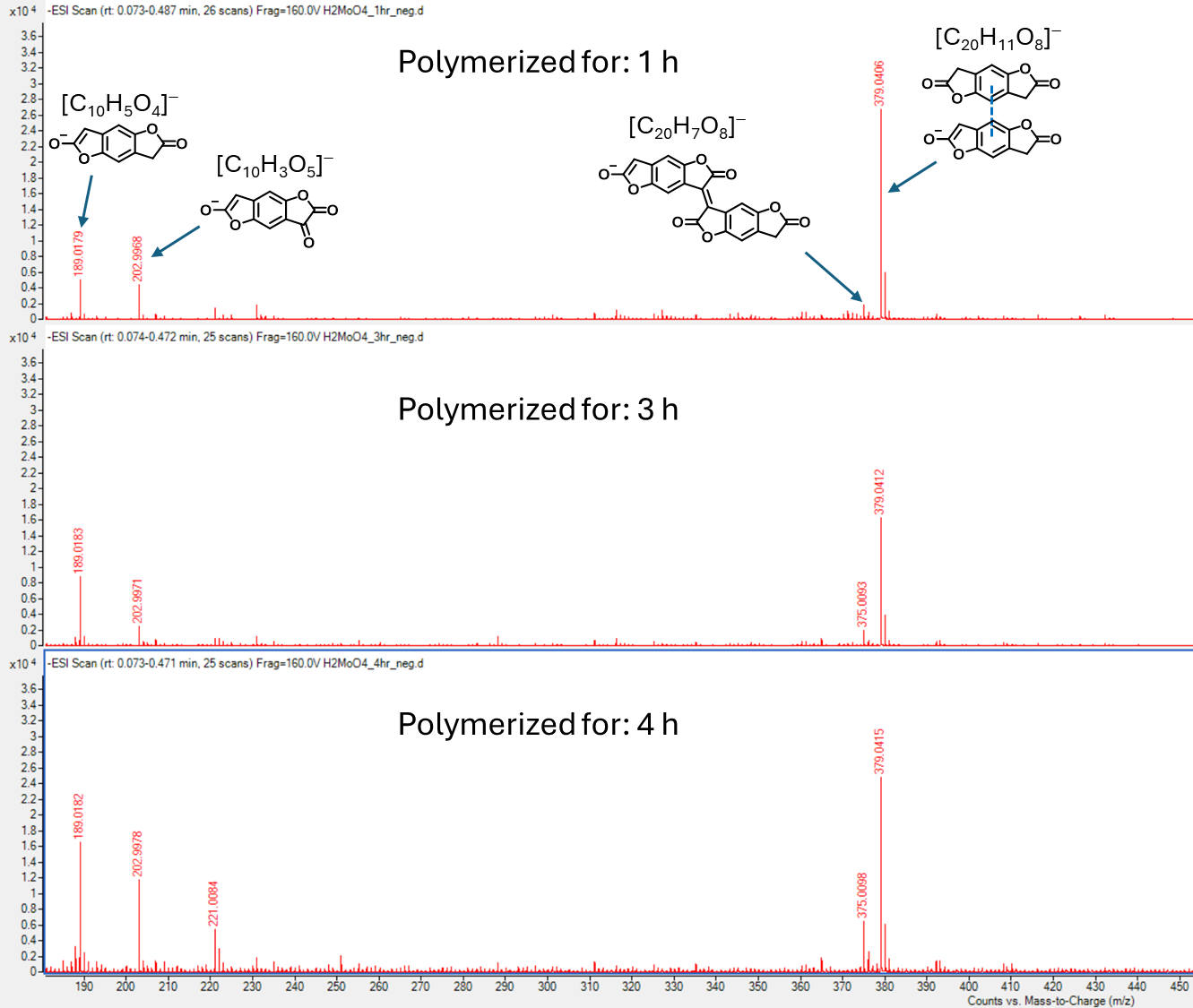


**Figure S19** ESI-MS of the mixtures from the polymerization with 0.001 eq H_2_MoO_4_.

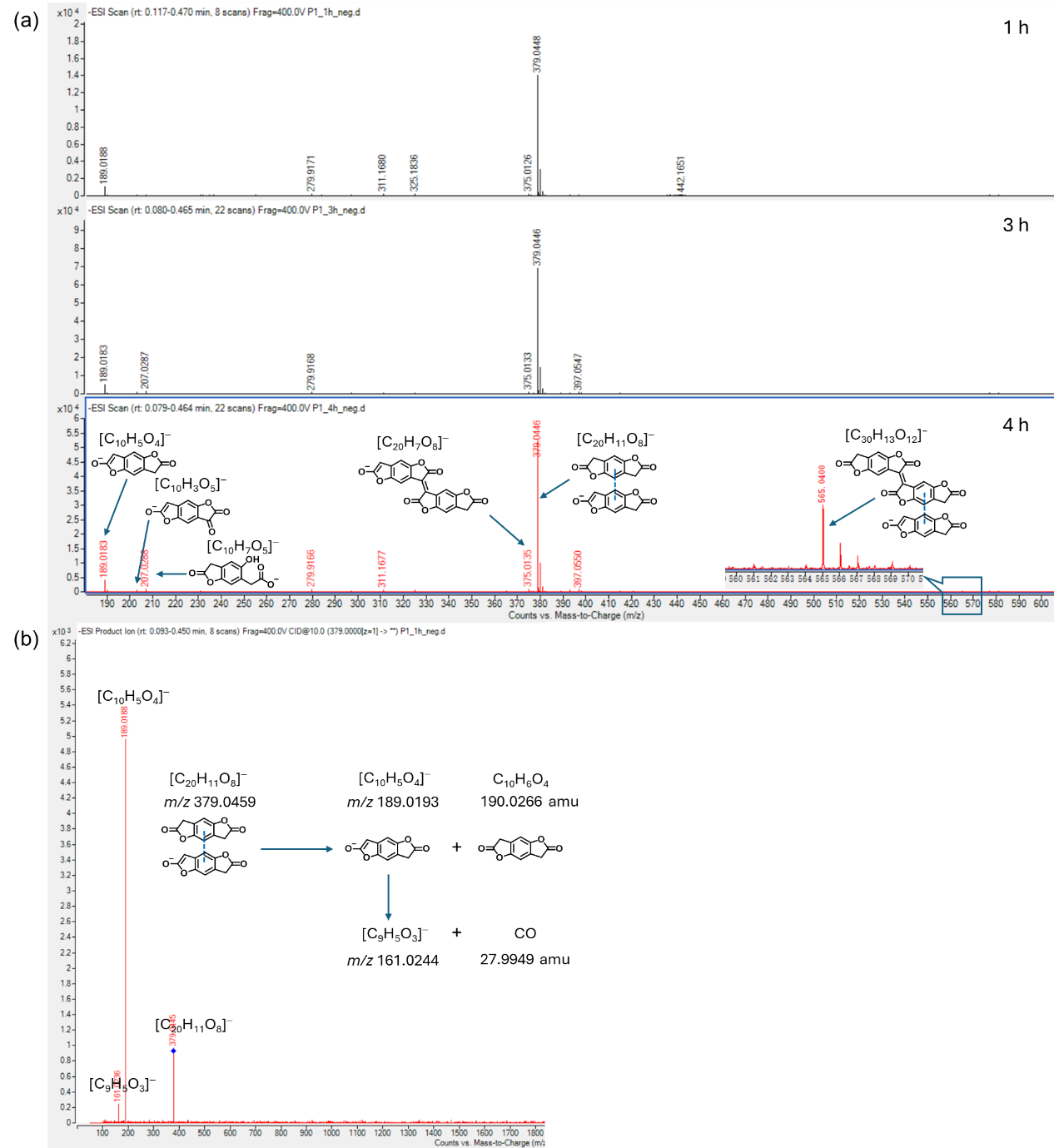


**Figure S20** (a) ESI-MS of the mixtures from the polymerization with 0.1 eq H_2_MoO_4_. (b) CID ESI-MS product ion scan of *m/z* 379.0445 reveals it to be a CTC of two monomers. The ESI-MS results from 0.1 eq H_2_MoO_4_ show similar results to polymerizations with 0.001 eq MoO_3_ and H_2_MoO_4_ that BDF monomer, the diketone intermediate, the dimer and the CTC of two BDF were detected. In addition, the CTC formed by dimer and BDF (*m/z* 565.0440) was observed in the enlarged spectrum within the *m/z* range of 560-570.

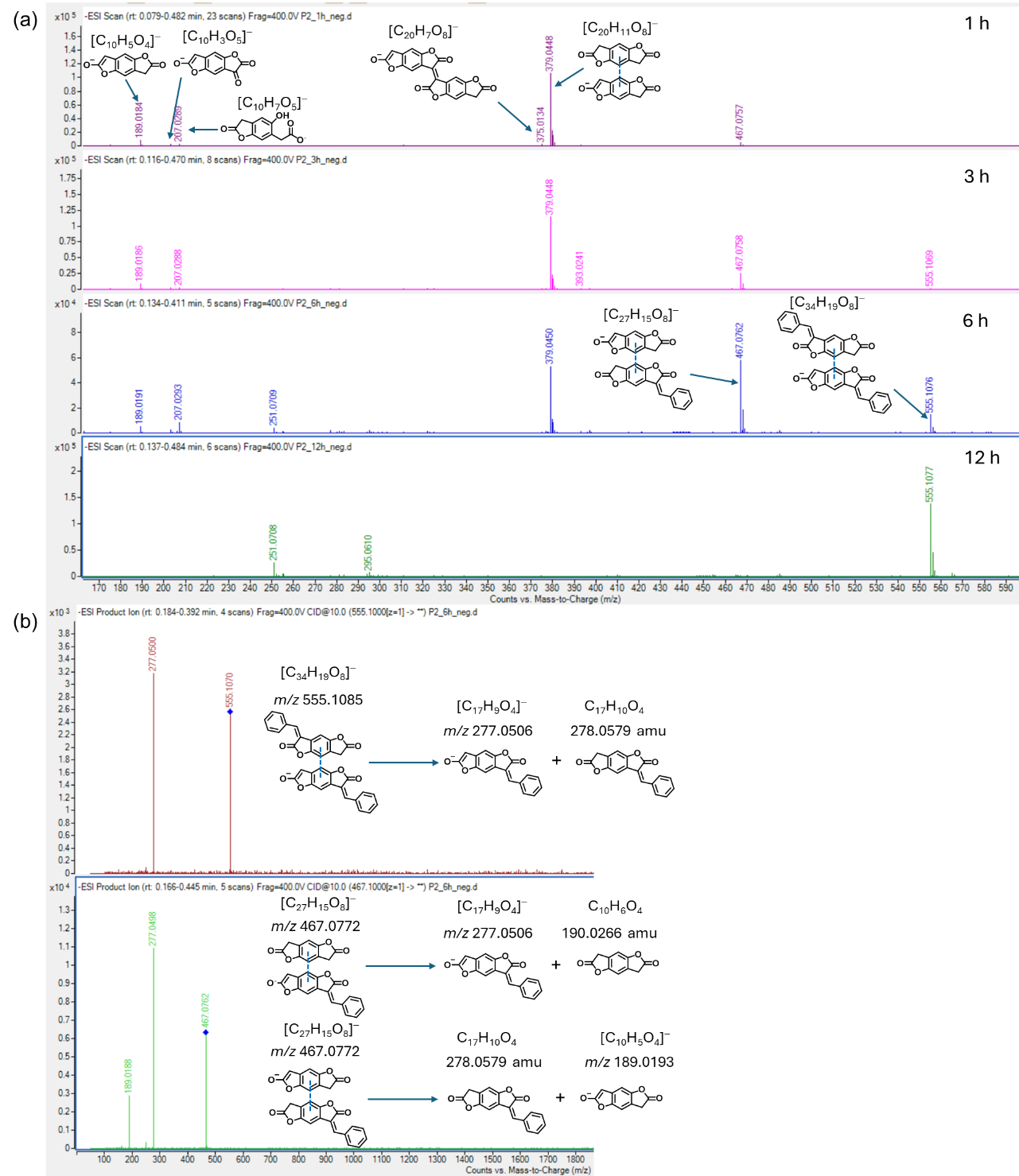


**Figure S21** (a) ESI-MS of the mixtures from the polymerization with 0.1 eq H_2_MoO_4_ and 1 eq PBN added. (b) CID ESI-MS product ion scan of *m/z* 467.0762 and 555.1070. It can be seen that the diketone intermediate and dimer were also detected in the early stage of polymerization, but no spin adducts of BDF monomer and PBN were observed.^[7]^ Finally, the monomer was consumed and the condensation product of BDF monomer and PBN (*m/z* 277.0506) was observed.


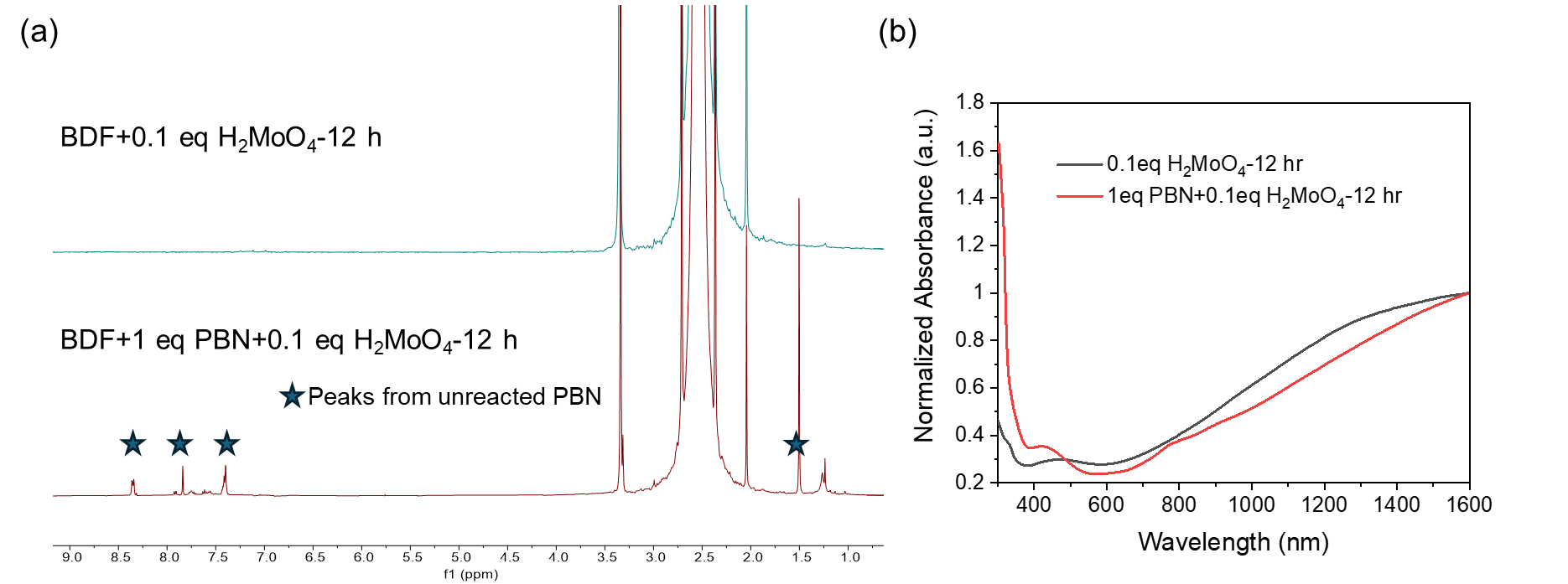


**Figure S22** (a) ^1^H NMR, (b) UV–Vis–NIR absorption spectra of the mixtures obtained from the polymerization with and without PBN added. The monomer was consumed in both two polymerizations after 12 h. However, there is still a large amount of unreacted PBN residue in the polymerization with PBN added. The UV–Vis–NIR absorption spectra of mixture obtained from solution polymerization with PBN added shows similar absorption curve of n-PBDF, which shows strong polaron and bipolaron absorption bands in the NIR region, suggesting the polymerization reaction proceeds relatively smoothly.


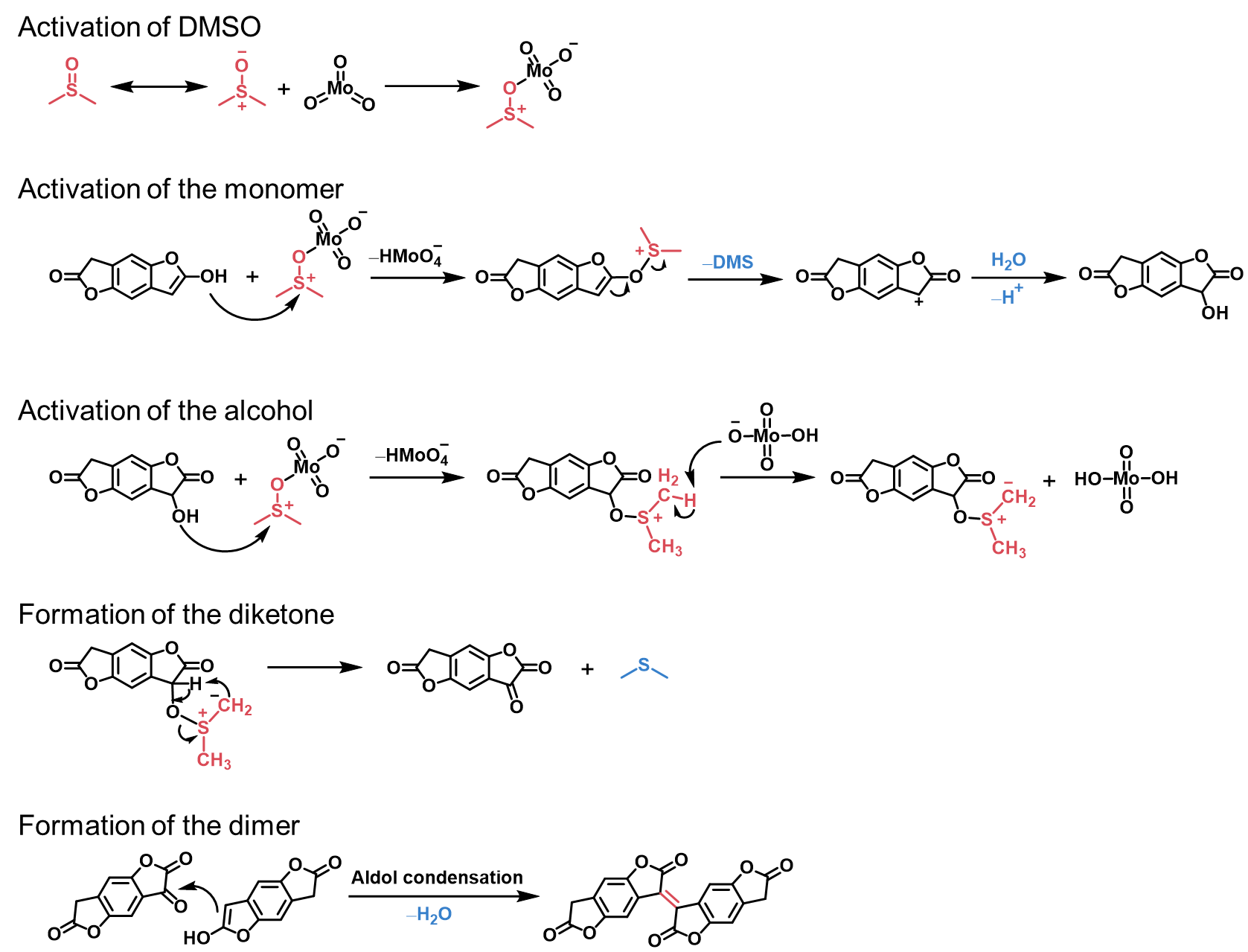


**Figure S23** The proposed mechanisms for the MoO_3_-DMSO complex mediated dimerization process.


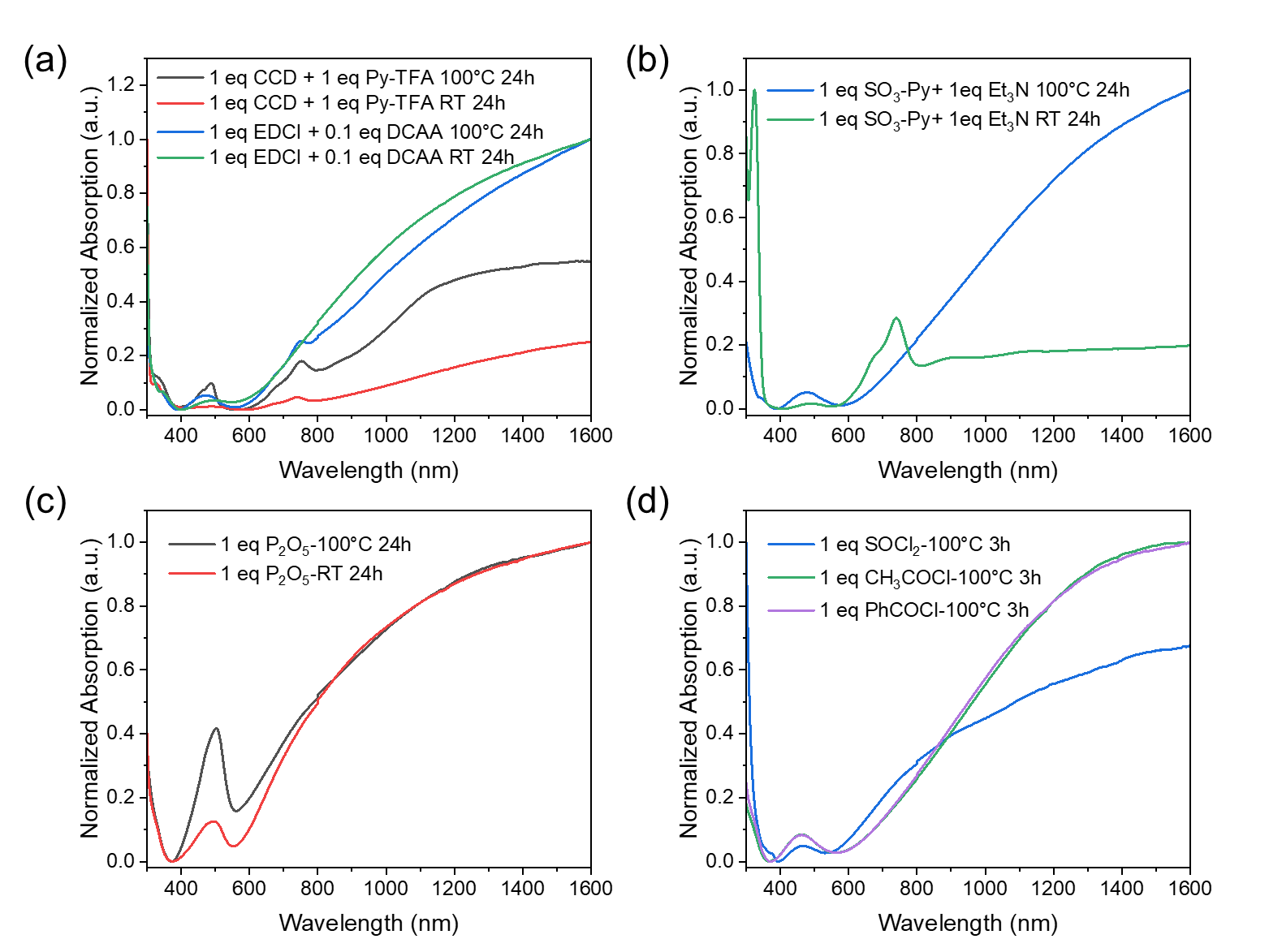


**Figure S24** UV-Vis-NIR spectra of the BDF polymerizations by DMSO oxidation activated by (a) CCD and EDCI, (b) SO_3_-Py, (c) P_2_O_5_, and (d) SOCl_2_, CH_3_COCl, and PhCOCl. The above UV-Vis-NIR spectra exhibit characteristic absorption curve of n-PBDF, in which strong polaron and bipolaron absorption bands are observed in the NIR region, indicating that both DCC, EDCI, SO_3_-Py, P_2_O_5_, SOCl_2_, CH_3_COCl, and PhCOCl can activate DMSO and trigger the oxidation polymerization of BDF monomer, although these polymerization conditions have not been optimized.

Based on the above results, we propose a general DMSO oxidation pathway of BDF monomer by using activating agents, as shown in **Figure S25a**. Under the activation of the electrophilic reagent, BDF monomer nucleophile attacks the sulfur atom, then further undergoes elimination and loses dimethyl sulfide (DMS) to form the diketone product, thereby achieving the oxidation process. Some examples of electrophilic reagents capable of activating DMSO are summarized in **Figure S25b**, these reagents may be capable of triggering the polymerization of BDF monomers, although some of them were not explored in this study. However, stoichiometric amounts of these activation reagents were required in all examples except for MoO_3_ in this study, as they are consumed and cannot be recycled.


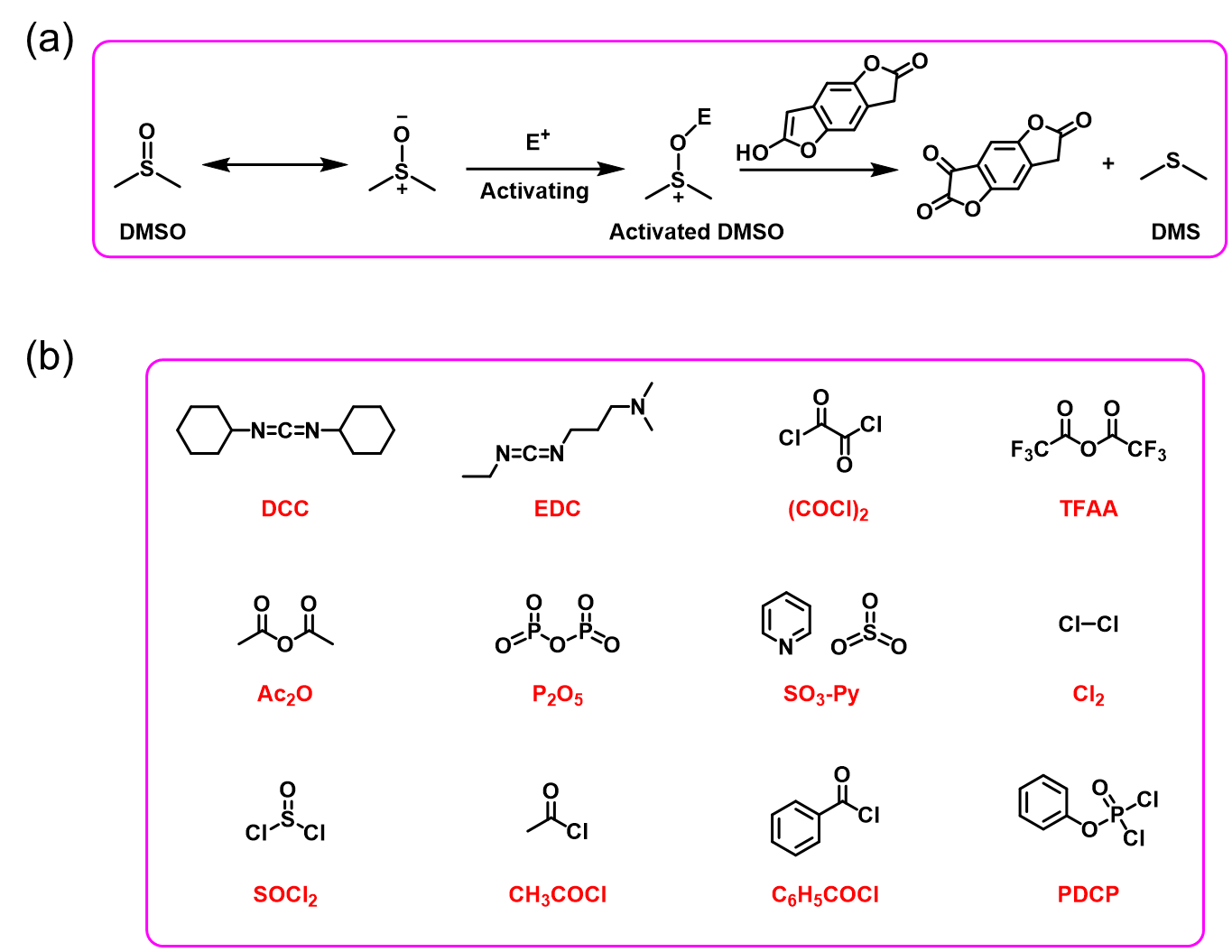


**Figure S25** (a) The general DMSO oxidation pathway of BDF monomer by using activating agents. (b) Some examples of electrophilic reagents capable of activating DMSO.

**Figure S26** The proposed possible pathways involved in the “initiation period” of MoO_3_ catalyzed polymerization.

1. **Scale-up synthesis of n-PBDF ink by using ppm level MoO_3_**

Scale-up synthesis of n-PBDF ink by using 100 ppm MoO_3_:

The BDF monomer (5.0 g, 26.30 mmol, 1 eq) was dissolved in DMSO (1 L) at 100 ℃ under nitrogen atmosphere. Then, 3.8 mL of 0.1 mg mL^−1^ MoO_3_ solution in DMSO (0.38 mg, 0.00263 mmol, 0.0001 eq, 100 ppm) was added into the reaction system. The mixture was stirring at 100 ℃. During the polymerization process, Thin Layer Chromatography (TLC) and ^1^H NMR were utilized to monitor the monomer conversion until the monomer was completely polymerized (**Figure S27**). The total reaction time was 60 hours. After the solution was cooled to RT, the medium viscosity n-PBDF ink was obtained.


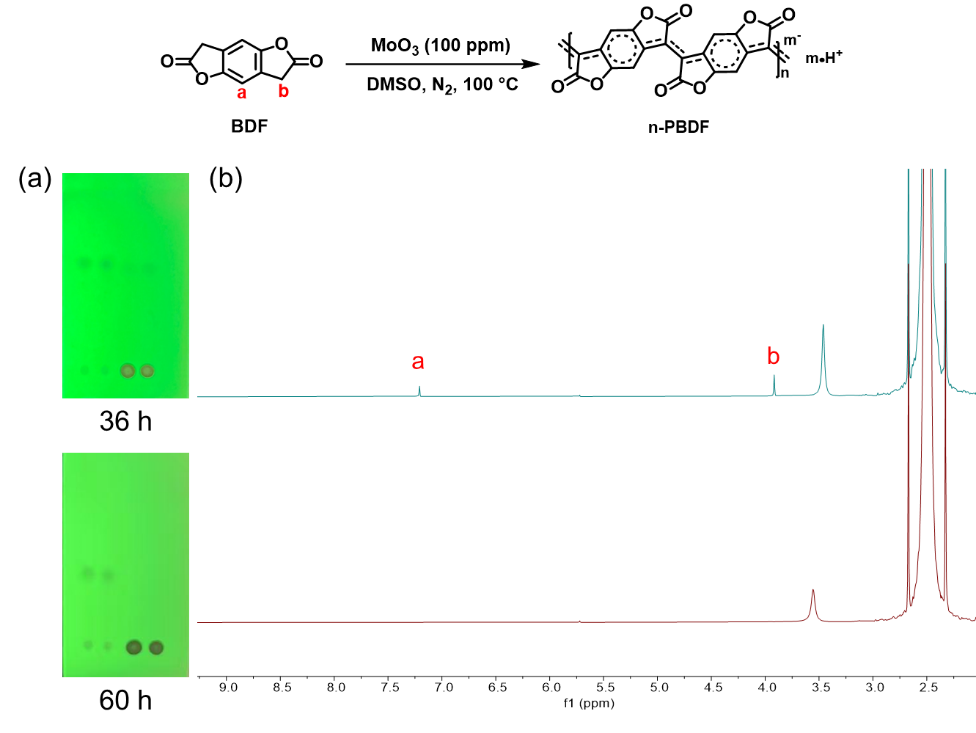


**Figure S27** Monitoring of the scale-up polymerization (1 L) of n-PBDF catalyzed by 100 ppm MoO_3_. (a) TLC of BDF monomer (the left two dots) and reaction mixtures (the right two dots) at 36 h and 60 h (b) ^1^H NMR spectra of reaction mixtures at 36 h and 60 h.

Scale-up synthesis of n-PBDF ink by using 10 ppm MoO_3_:

The BDF monomer (5.0 g, 26.30 mmol, 1 eq) was dissolved in DMSO (1 L) at 100 ℃ under nitrogen atmosphere. Then, 0.38 mL of 0.1 mg mL^−1^ MoO_3_ solution in DMSO (0.038 mg, 0.000263 mmol, 0.00001 eq, 10 ppm) was added into the reaction system. The mixture was stirring at 100 ℃ (**Figure S28**). During the polymerization process, ^1^H NMR was utilized to monitor the monomer conversion until the monomer was completely polymerized (**Figure S29**). After 9 days of polymerization, the reaction mixture became very viscous, but ^1^H NMR spectra shows that the monomer was not completely polymerized. To avoid the mixture forms gel state, 0.25 L DMSO was added, the mixture continued stirring for 1 day. As shown in **Figure S29**, the monomer was completely polymerized in 10 days. After the solution was cooled to RT, the medium viscosity n-PBDF ink was obtained.


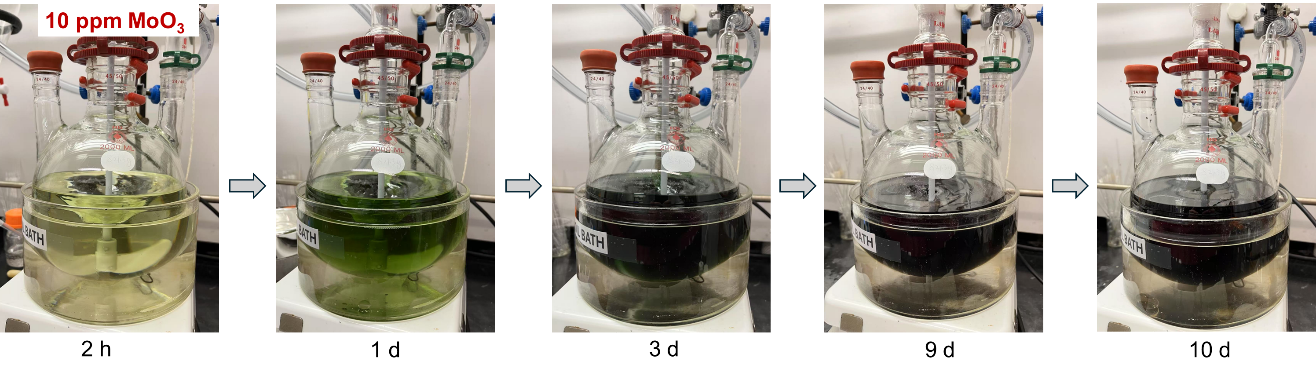


**Figure S28**. Scale-up polymerization (1 L) of n-PBDF catalyzed by 10 ppm MoO_3_.


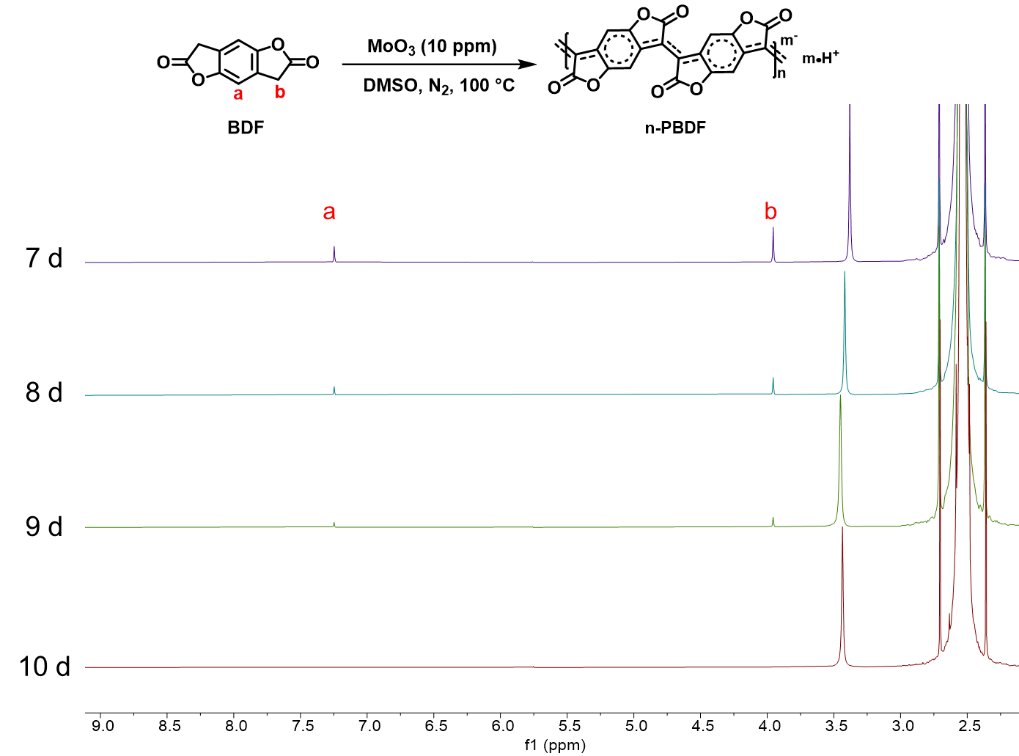


**Figure S29** ^1^H NMR Monitoring of the scale-up polymerization (1 L) of n-PBDF catalyzed by 10 ppm MoO_3_.

1. **Generalization and application of MoO_3_ catalyzed polymerization**

**Synthesis of Polymer n-PBDT**

The 3,7-dihydrobenzo[1,2-b:4,5-b']dithiophene-2,6-dione (BDT) monomer (50.0 mg, 0.2249 mmol, 1 eq) was dissolved in DMSO at 100 ℃ under nitrogen atmosphere. Then, 0.324 mL of 0.1 mg mL^−1^ MoO_3_ solution in DMSO (0.0324 mg, 0.0002249 mmol, 0.001 eq, 1000 ppm) was added into the reaction system. The total volume of DMSO is 10 mL. The mixture was stirring for 12 hours until BDT monomer conversion was complete (checked by ^1^H NMR).

The n-PBDT polymer can continue to propagate by adding the second batch of BDT monomer: 5 mL of 5 mg mL^−1^ BDT monomer solution (25 mg, predissolved in 5 mL DMSO at 100 ℃) was added into 5 mL of the above-mentioned n-PBDT prepolymer solution at 100 ℃, and the newly added BDT monomer was rapidly and completely polymerized after 1 h, as shown in **Figure S30**.


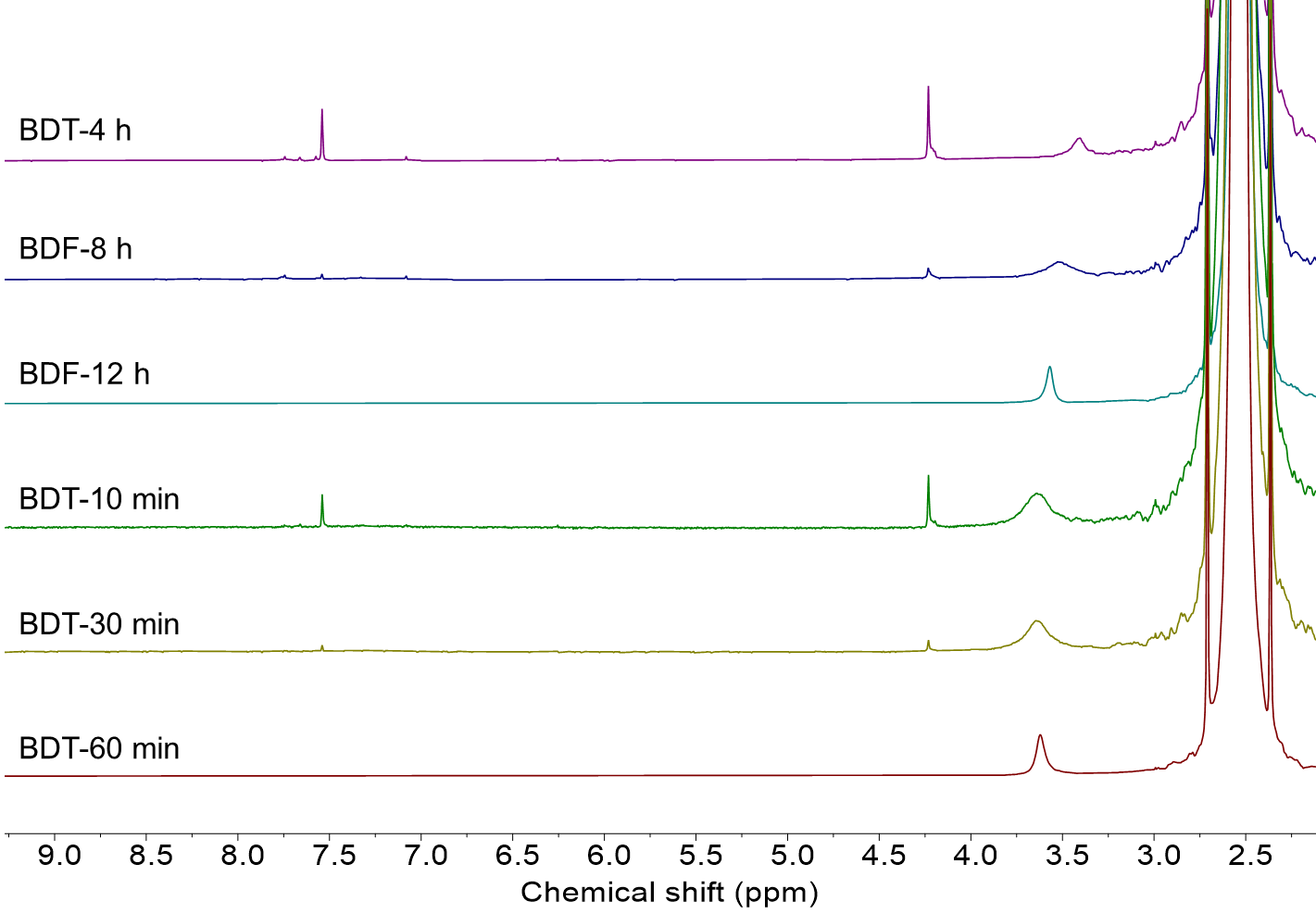


**Figure S30** ^1^H NMR monitoring of the polymerization process of n-PBDT and its “monomer-addition” experiment.

**Synthesis of block copolymers by MoO_3_ catalyzed polymerization**

**Synthesis of n-PBDF-*b*-PBDT**: the prepolymer n-PBDF was synthesized following the procedure that described in **Section 3**. BDF monomer (50.0 mg, 0.2630 mmol, 1 eq) was dissolved in DMSO at 100 ℃ under nitrogen atmosphere. Then, 0.380 mL of 0.1 mg mL^−1^ MoO_3_ solution in DMSO (0.0380 mg, 0.000263 mmol, 0.001 eq, 1000 ppm) was added into the reaction system. The total volume of DMSO is 10 mL. The mixture was stirring for 15 hours until BDF monomer conversion was complete (checked by ^1^H NMR). Then, 5 mL of 5 mg mL^−1^ BDT monomer solution (25 mg, predissolved in 5 mL DMSO at 100 ℃) was added into 5 mL of the above-mentioned n-PBDF prepolymer solution at 100 ℃, the mixture was continued stirring for 1 h until BDT monomer conversion was complete (checked by ^1^H NMR). After the mixture was cooled to room temperature, the block copolymer n-PBDF-*b*-PBDT was obtained as a medium viscosity ink.


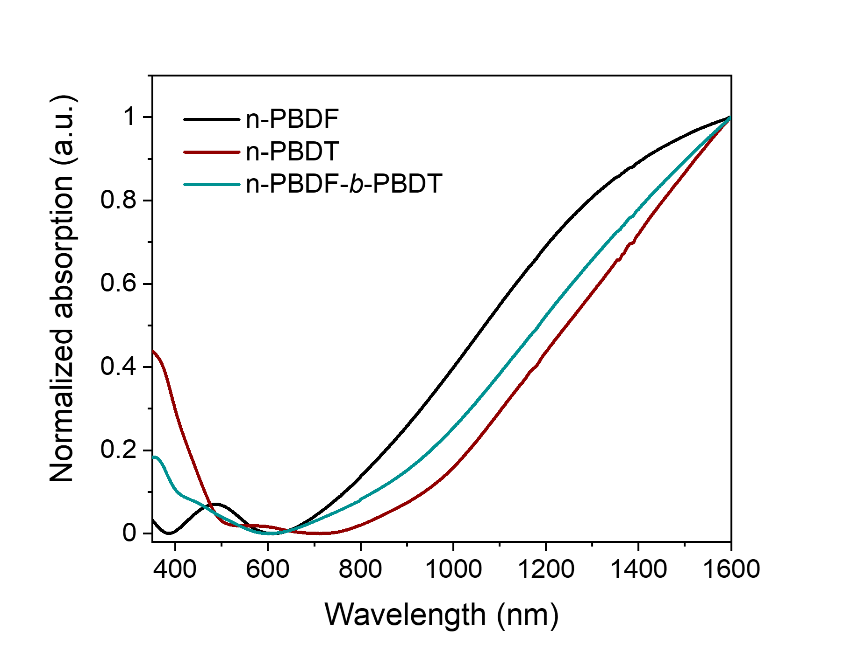


**Figure S31** UV-Vis NIR spectra of n-PBDF, n-PBDT and n-PBDF-*b*-PBDT in DMSO.

**Table S6** Characterization of n-PBDF and block copolymer n-PBDF-*b*-PBDT

| Polymer | Hydrodynamic diameter (nm) | Conductivity (S cm^−1^) |  |
| --- | --- | --- | --- |
| n-PBDF | 70.6 ± 0.5 | 2075 ± 130 |  |
| n-PBDT | 52.7 ± 1.7 | 0.082 ± 0.004 |  |
| n-PBDF-*b*-PBDT | 82.0 ± 1.2 | 436 ± 52 |  |
| n-PBDF & n-PBDT mixture*^a^* | 60.9 ± 2.1 | 270 ± 26 |  |

*^a^*The mixture was prepared by mixing the above-mentioned n-PBDF and n-PBDT in equal volumes.


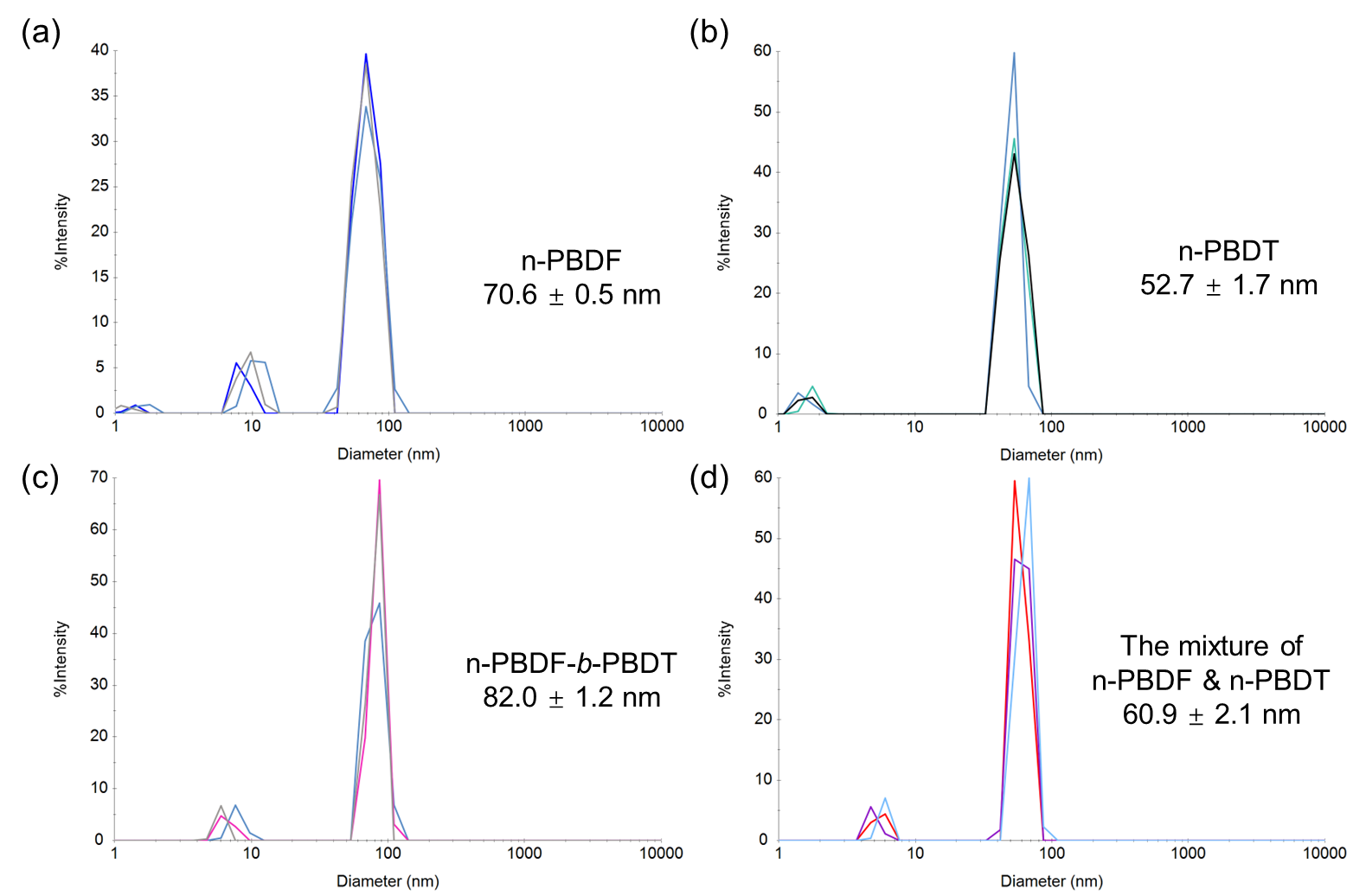


**Figure S32** DLS analysis of hydrodynamic diameter distribution of (a) n-PBDF, (b) n-PBDT, (c) block copolymer n-PBDF-*b*-PBDT, (d) The mixture of n-PBDF and n-PBDT.

**Synthesis of n-PBDT-*b*-PBDF**: the prepolymer n-PBDT was synthesized following the above-mentioned procedure. Then, 5 mL of 2.5 mg mL^−1^ BDF monomer solution (12.5 mg, predissolved in 5 mL DMSO at 100 ℃) was added into 5 mL of the above-mentioned n-PBDT prepolymer solution at 100 ℃, the mixture was continued stirring for 3 h until BDF monomer conversion was complete, as shown in **Figure S33**. After the mixture was cooled to room temperature, the block copolymer n-PBDT-*b*-PBDF was obtained as a gel solution and diluted by DMSO to afford the medium viscosity ink.


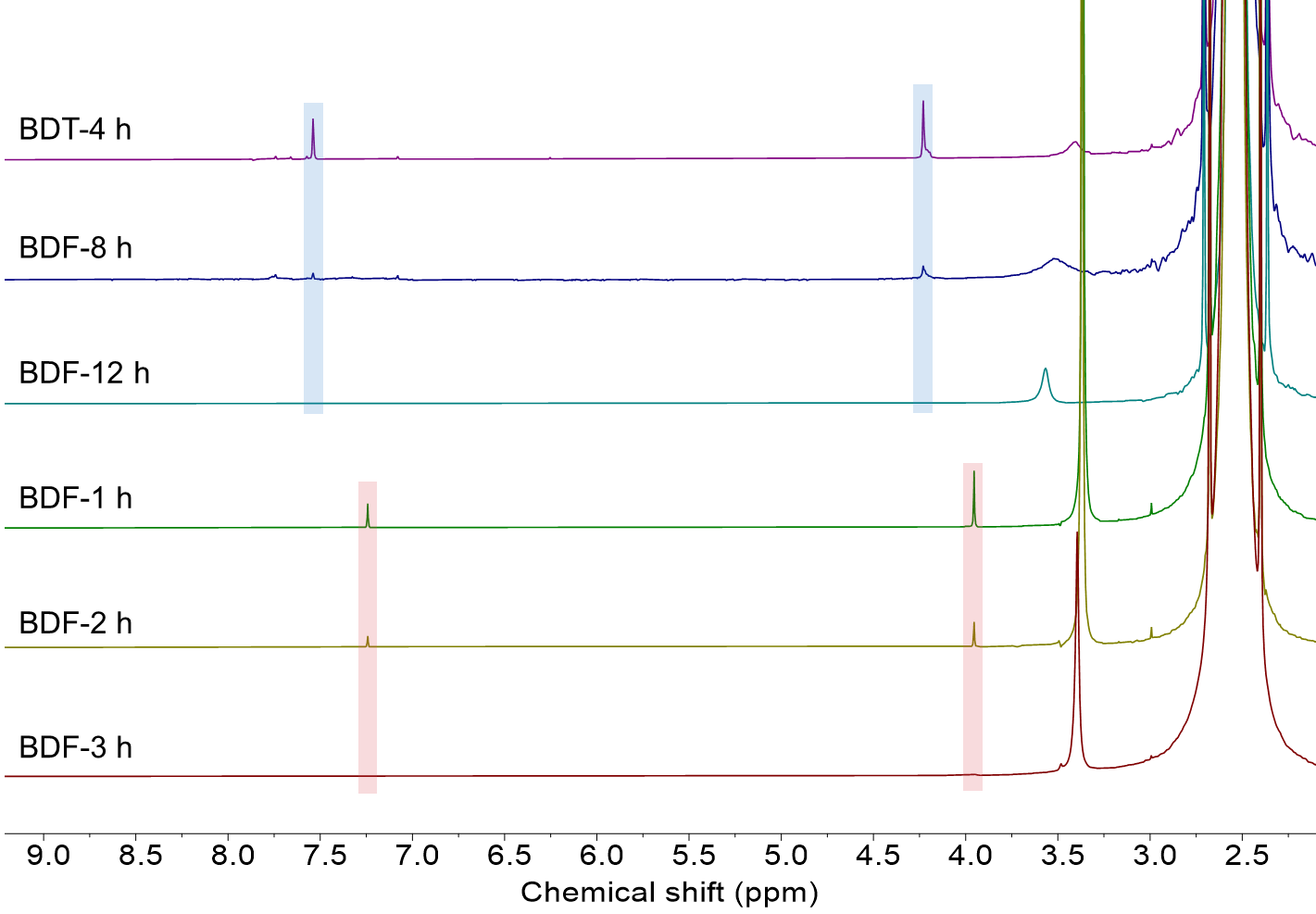


**Figure S33** ^1^H NMR monitoring of the polymerization process of block copolymer n-PBDT-*b*-PBDF. The proton peaks of BDF and BDT are marked as red and blue, respectively.

**References**

[1] T. Lei, J. H. Dou, X. Y. Cao, J. Y. Wang, J. Pei, *J. Am. Chem. Soc.* **2013**, *135*, 12168-12171.

[2] H. Tang, H. Cai, H. Zhao, Z. Liu, R. Tan, F. Huang, *CCS Chemistry* **2023**, *5*, 2534-2544.

[3] G. Liu, H. H. Hsu, S. Samal, W. J. Lee, Z. Ke, L. You, B. M. Savoie, J. Mei, *Angew. Chem. Int. Ed.* **2025**, *64*, e202418668.

[4] C. J. W. Richard N. Hider, *J. Chem. Soc., Dalton Trans.* **1984**, 495-500.

[5] a) Z. Ke, A. Abtahi, J. Hwang, K. Chen, J. Chaudhary, I. Song, K. Perera, L. You, K. N. Baustert, K. R. Graham, J. Mei, *J. Am. Chem. Soc.* **2023**, *145*, 3706-3715; b) J. Hwang, Q. Zhao, M. Ahmed, A. C. Yakisan, M. F. Espenship, J. Laskin, B. M. Savoie, J. Mei, *Angew. Chem. Int. Ed.* **2024**, *63*, e202401465; c) Z. Ke, J. Chaudhary, L. Q. Flagg, K. N. Baustert, A. O. Yusuf, G. Liu, L. You, K. R. Graham, D. M. DeLongchamp, J. Mei, *Adv. Funct. Mater.* **2024**, *34*, 2400255; d) J. Hwang, X. Ni, M. F. Espenship, K. Tang, J. Zhang, A. Basu, S. Kuila, S. Barlow, S. R. Marder, J. L. Bredas, J. Laskin, J. Mei, *J. Am. Chem. Soc.* **2025**, *147*, 19372-19379.

[6] Y. Huang, Y. Yu, R. Hu, B. Z. Tang, *J. Am. Chem. Soc.* **2024**, *146*, 14685-14696.

[7] H. Tang, Y. Liang, C. Liu, Z. Hu, Y. Deng, H. Guo, Z. Yu, A. Song, H. Zhao, D. Zhao, Y. Zhang, X. Guo, J. Pei, Y. Ma, Y. Cao, F. Huang, *Nature* **2022**, *611*, 271-277.
